# Research Progress in Shape-Control Methods for Wire-Arc-Directed Energy Deposition

**DOI:** 10.3390/ma17235704

**Published:** 2024-11-21

**Authors:** Jie Wang, Bo Zhao, Yuanlin Liu, Junjie Zhao, Guangyu Ma

**Affiliations:** 1 School of Material Science and Engineering, Shandong Jianzhu University, Jinan 250101, China; 202210103011@stu.sdjzu.edu.cn (J.W.); 2023105125@stu.sdjzu.edu.cn (Y.L.); zhaojj23@sdjzu.edu.cn (J.Z.); 2023105108@stu.sdjzu.edu.cn (G.M.); 2 Research Institute of Materials Reliability for Advanced Equipments, Shandong Jianzhu University, Jinan 250101, China

**Keywords:** wire-arc-directed energy deposition, shape control, wire-arc additive manufacturing, regulation of process parameters, deposition path optimization, auxiliary energy and mechanical fields, integrated additive–subtractive manufacturing

## Abstract

Wire-arc-directed energy deposition (WA-DED) stands out as a highly efficient and adaptable technology for near-net-shaped metal manufacturing, with promising application prospects. However, the shape control capability of this technology is relatively underdeveloped, necessitating further refinement. This review summarizes the latest advancements in the shape control of WA-DED technology, covering four pivotal areas: the regulation of various process parameters, optimization of the deposition paths, control through auxiliary energy and mechanical fields, and synergy between additive and subtractive manufacturing approaches. Firstly, this review delves into the influence of deposition current, travel speed, wire feed speed and other parameters on the forming accuracy of additively manufactured parts. This section introduces control strategies such as heat input and dissipation management, torch orientation adjustment, droplet behavior regulation, and inter-layer temperature optimization. Secondly, various types of overlap models and techniques for designing overall deposition paths, which are essential for achieving desired part geometries, are summarized. Next, auxiliary fields for shape and property control, including magnetic field, ultrasonic field, and mechanical field, are discussed. Finally, the application of milling as a subtractive post-process is discussed, and the state-of-the-art integrated additive-subtractive manufacturing method is introduced. This comprehensive review is designed to provide valuable insights for researchers who are committed to addressing the forming defects associated with this process.

## 1. Introduction

Additive manufacturing (AM), also known as 3D printing, has emerged as one of the most vibrant research areas in recent years [1,2]. AM technology enables the rapid prototyping of solid parts and complex structures with high efficiency, mold-free production, and near-net-shape manufacturing, thanks to its process during which materials are deposited and stacked layer by layer [3]. In the field of metalworking, additive manufacturing (AM) predominantly features two methods: powder bed fusion (PBF) and directed energy deposition (DED). PBF operates by selectively melting or sintering powder particles with a laser, layer by layer, within a powder bed [4]. DED entails melting raw materials, whether in powder or wire form, through the application of heat, subsequently transporting them onto a substrate to create a small molten pool and continuously depositing them in successive layers. DED’s higher deposition speed allows the production of larger components than PBF [5]. According to ASTM F3413-2019 [6], parts manufactured with DED under deposition conditions typically demonstrate better static and dynamic mechanical properties than those produced by PBF. However, DED generally offers less manufacturing precision. DED is versatile and capable of depositing a wide range of metallic materials, including various types of steels, alloys, intermetallics, ceramics, and composites [7,8,9,10]. Additionally, during the deposition process using powder bed fusion (PBF), the melt pool does not capture all the powder, which can reduce deposition efficiency. Moreover, some powder is ejected into the surrounding environment, posing potential risks to operators and the environment. In contrast, the use of wire in DED technology reduces operational risks for workers and makes the process more environmentally friendly [11]. However, DED technology based on wire can produce intense arc light and fumes, requiring workers to take necessary protective measures. In terms of equipment and material costs, industrial-grade PBF systems typically cost over USD 100,000, while wire-arc-based DED systems can be kept under USD 2000 [12,13,14]. The price of wire materials is relatively lower compared to that of powder materials. For example, the price of wire Ti-6Al-4V is about 125 USD/kg, while the price of powder can be as high as 300 USD/kg; the price of wire IN625 is about 52 USD/kg, while the price of powder is about 83 USD/kg. Furthermore, studies have shown that the cost of manufacturing basic components using PBF technology is five times that of using DED technology [3,15,16,17].

Depending on the heat source, DED technology can be divided into laser-directed energy deposition, electron beam-directed energy deposition, and weld arc-directed energy deposition [18]. Laser-directed energy deposition (L-DED) technology stands out for its focused energy beam and small spot size, enabling the production of intricate small-scale components like biomedical scaffolds [19]. This technology goes by several names, such as selective laser melting (SLM), selective laser sintering (SLS), and laser additive manufacturing (LAM). It is worth noting that selective laser sintering (SLS) traditionally pertains to powder sintering rather than melting. L-DED’s production rate is relatively modest, ranging from 150 to 200 g per hour, with an energy efficiency between 20% and 50% [15,20,21], posing challenges for the fabrication of large-sized parts. Electron beam directed energy deposition (EB-DED) requires a vacuum for operation, which restricts the size of the parts to the dimensions of the vacuum chamber. Its deposition speed is 600 to 800 g per hour. However, it is susceptible to contamination, with risks of dust, gases, and other potential impurities being incorporated into the parts during deposition. Compared with other metal additive manufacturing technologies, wire-arc-direct energy deposition (WA-DED) has the characteristics of high deposition efficiency and material utilization, low manufacturing cost, and easy manufacturing of large parts [22]. Its deposition efficiency can reach up to 14 kg per hour [23], with an energy efficiency ranging from 54% to 88% [24,25], Additionally, it boasts a high cooling rate, varying within the range of 100 to 1000 degrees Celsius per second [26].

The history of this technology date back to 1925 when Ralph Baker succeeded in depositing metal and fabricating metal vessel walls by manipulating the arc between an electrode and a metal plate [27]. Over the course of the subsequent century, WA-DED technology has undergone various names, including shape welding (SW), shape melting (SM), rapid prototyping (RP), solid freeform fabrication (SFF), shape metal deposition (SMD), 3D welding, and weld arc additive manufacturing [28]. Fifty-eight years after Ralph Baker’s pioneering patent, Kussmaula employed the “Shape Welding” technique to craft solid components, hinting at the technology’s impending commercial viability [29]. Subsequently, advancements in materials science, computing, and automation trickled into manufacturing. However, it was not until the 1990s that the academic community recognized arc welding as a viable method for rapid prototyping [26].

Fifty-eight years following Ralph Baker’s groundbreaking patent, Kussmaul and his team utilized the “Shape Welding” technique to fabricate solid components, signaling the technology’s potential for commercial application. Subsequent progress in materials science, computing, and automation gradually transformed manufacturing. Nowadays, as the demand for lightweight, high strength-to-weight ratio, large scale, and monolithic metal parts increases in aerospace, automotive, and ship industries, WA-DED is being extensively and intensively researched and developed [30,31]. Currently, parts that can be produced using WA-DED technology include impeller shafts [32], aircraft wing beams, satellite panels, fuel tanks, and more [33]. Also, large-scale components, such as an airplane’s nose cone with a diameter of 190 mm fabricated through WA-DE, have been undergoing testing in a transonic wind tunnel to explore the viability of utilizing WA-DED technology for the production of entire aircraft [34]. These practical application examples all demonstrate the potential of WA-DED technology.

**Figure 1:**
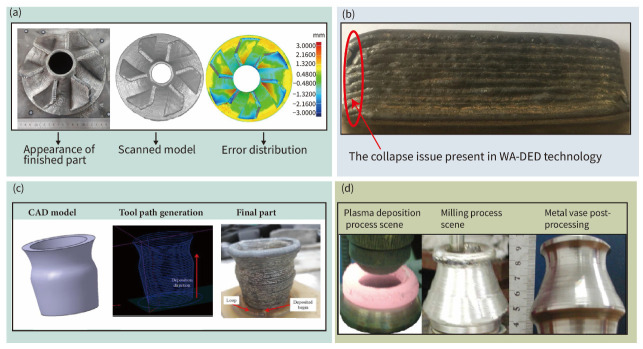
Forming accuracy of parts related to WA-DED. (**a**) The standard deviation of a typical turbine part is 0.95 mm; (**b**) Deposition layer collapse phenomenon; (**c**) The process of manufacturing parts using a 4-axis MPAW-based WAAM system; (**d**) Metal direct prototyping by using hybrid plasma deposition and milling [38,40,42,43].

However, despite its rapid growth, the technology is still in the emerging stage. The ability to control the “shape” of additively manufactured structures has always been a hot topic and challenge in recent years [35]. Researchers have proposed various methods to regulate the process of melting, flow and solidification of the deposited metal during WA-DED process, which includes the control of process parameters, optimization of the deposition sequence or path, the addition of energy and mechanical field assistance. These methods have improved the WA-DED technology’s forming accuracy and surface quality to a certain extent, but they are insufficient to meet the requirements of high-accuracy metal parts and components. Further research and development are needed [36,37]. Figure 1 displays recently produced components utilizing WA-DED technology. Notably, a 200 mm turbine component crafted via GMA-DED technology exhibits a manufacturing standard deviation of 0.95 mm (as shown in Figure 1a) [38]. The illustration (Figure 1b) further shows that certain sections of the fabricated component possess a precision error as high 3 mm. In contrast, GB/T 1184-1996 stipulates a minimum standard deviation requirement of 0.8 mm for straightness and flatness in manufactured parts, which pertains specifically to planes without specific precision demands [39]. Additionally, problems such as collapse in WA-DED and imperfections at the deposition initiation and termination points remain unresolved [40]. Some studies have revealed that the height and width discrepancies between the deposition’s start and end points, compared to the middle section, hover around 8% [41]. Figure 1c illustrates that parts fabricated using four-axis MPAW-based WAAM technology exhibit a relative error ranging from 10% to 17% when compared to CAD models, indicating ample scope for enhancement in shaping accuracy control [42]. For the burgeoning needs of manufacturing high-performance, large-scale, and monolithic metal parts, hybrid additive and subtractive manufacturing is needed to produce high-precision components with accuracy measured in micrometers, which is depicted in Figure 1d and will be further discussed in Section 6 of this review [43].

Based on the recent research progress in WA-DED technology in controlling the forming quality of parts, this paper initially discusses the characteristics of WA-DED technology and the reasons for the poor forming quality, and subsequently systematically summarizes the main schemes for improving the forming quality of parts from four aspects: process parameter controlling, path optimization, energy or mechanical field assistance, and the hybrid additive-subtractive technologies. This paper further explores the mechanism of these control schemes and anticipates advancements in forming quality control by WA-DED technology.

## 2. Brief Background of WA-DED and Its Shape Control Issues

**Figure 2:**
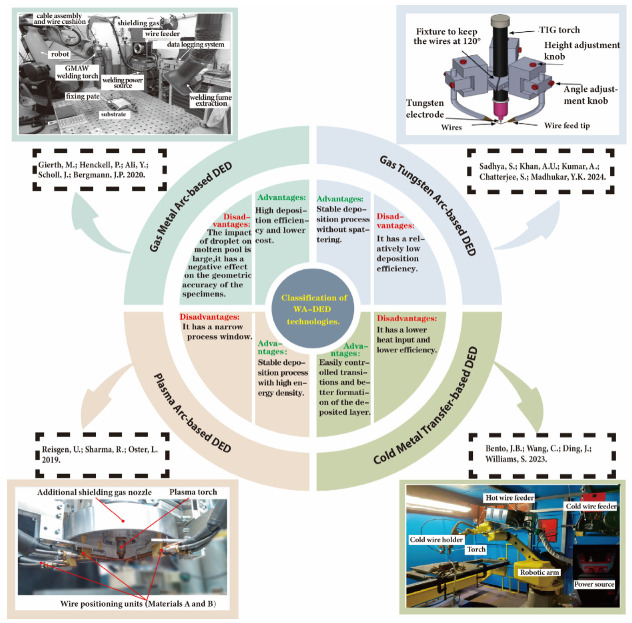
Typical WA-DED technologies and their advantages and disadvantages [51,52,53,54].

The WA-DED technology is based on conventional or classic arc welding techniques [44]. It uses welding wire as the raw material and an electric or plasma arc as the heat source. After the welding wire melts, it is transferred and deposited layer by layer on the substrate along a predetermined path, building up from linear to planar and then to volumetric construction. This process thereby accumulates material to form a metal component that meets the specified dimensions and manufacturing requirements [45]. WA-DED technology can be classified based on the classical arc welding techniques used. Currently, the mainstream arc welding processes used for WA-DED technology include gas metal arc welding (GMAW), gas tungsten arc welding (GTAW), plasma arc welding (PAW), and cold metal transfer (CMT), a welding technique that is a variant process of GMAW. Among these, GMA can be further divided into metal inert gas welding (MIG) and metal active gas welding (MAG). Each type of WA-DED technology has its characteristics. For instance, the GMA-based DED technology is also known as GMA-DED. Using consumable electrodes involves the consumption of the electrode itself during welding, has advantages in high deposition efficiency and low production cost [38]. The GTA-based DED technology features a stable deposition process with no spatter, non-consumable electrode methods involve feeding wire independently without consuming the electrode. Making it suitable for depositing materials such as aluminum alloys, nickel-based alloys, and titanium alloys [46]. PAW is applicable to nearly all metals, thus providing a broad material compatibility for PA-based DED technology, and it also benefits from high energy density. However, its reasonable parameter range is narrow, and the parameter matching is complex, which may limit its application [10,42]. In contrast, CMT-based DED technology features lower heat input, controllable droplet transfer, good forming characteristics, making it very suitable for depositing low-melting-point metals [47,48]. Figure 2 shows the classification and the advantages and disadvantages of typical WA-DED technologies. Apart from the aforementioned mainstream basic arc welding techniques, there are also welding processes such as tandem GMAW and variable polarity (VP) GTAW, which have been applied in WA-DED. For instance, WA-DED based on tandem GMAW offers higher deposition efficiency compared to single-wire GMAW, as this welding process integrates two welding wires within the same welding torch. On the other hand, WA-DED based on VP GTAW is mostly used for depositing aluminum alloys [49,50].

1. In general, the technical characteristics of WA-DED make it possible to deposit nearly all metallic materials [55,56]. However, during the deposition, several challenges may arise, such as excessive heat input, uneven heat distribution, weak confinement of the molten pool, and complex deposition path. If not properly controlled, these can lead to reduced forming accuracy and surface quality of deposition metal parts. Specifically, there are three main types of problems: During the deposition process, parts undergo repeatedly heating as they are built up layer by layer. This cycles of heating and cooling creates a complex temperature field. When thermal stress and phase transformation stress exceed the local yield strength of the part at a given temperature, macro deformation occurs, leading to reduced forming accuracy [57,58].
2. The deposition and forming process of molten metal undergoes the intricate metal transfer and weakly constrained molten pool flow and solidification, naturally resulting in undulating curved surfaces rather than smooth ones. This results in relatively high surface flatness or roughness, leading to inherent deviations from the expected part dimensions.
3. The layer-by-layer deposited beads are commonly under weak confinement, and deviations in the cooling rate, whether the too fast or too slow, can cause deviations in layer height and width from expectations, affecting the dimensional accuracy of the part.

The above content generally and briefly describes the reasons why WA-DED technology requires shape control and the specific factors affecting forming accuracy and surface quality. The mitigation measures currently adopted by most researchers to address the aforementioned issues are listed in Table 1. Some of the measures can be directly applied to industrial environments, such as the design and preheating of substrates, planning methods for deposition paths, and adjustments to electrical parameters, wire feed speed, travel speeds, and torch angles. These measures can be conveniently validated within industrial settings. However, the applicability of some measures still needs to be improved; for instance, the addition of external auxiliary energy fields and cooling equipment may reduce the overall flexibility of the system. When using hybrid additive and subtractive manufacturing techniques, it is necessary to assess the applicability based on the structure and geometric complexity of the part, as well as the accessibility of the tools [59]. It is worth noting that the mitigation measures outlined in Table 1 are brief summaries, and detailed explanations are provided in the following text.

## 3. Process Parameters Control Strategies

Extensive research has been conducted on the impact of various process parameters in WA-DED process, including deposition current, arc voltage, wire feed speed, travel speed (of the torch), deposition angle, droplet transfer behavior, inter-layer temperature, and so on. These parameters are meticulously studied because their optimal selection is fundamental to achieving good part formation.

### 3.1. Deposition Current, Arc Voltage, Travel Speed, and Wire Feed Speed

Electrical parameters have a direct impact on the accuracy of the deposition layer formation. Xiong [60] found that when depositing multi-layer single-pass wall-shaped structures based on GMA-DED, different degrees of collapse occurred in the appearance under high deposition currents of 250 A or 300 A, while good formation was achieved under lower current conditions (100–180 A). This was attributed to the fact that molten droplets at low deposition current had minimal impact on the molten pool and a small heat input, which reduced the likelihood of remelting the previously deposited layer. Hu et al. [61] established a three-dimensional transient fluid model to simulate the metal flow and solidification in the molten pool. The occurrence of defects at the deposition start point is attributed to the absence or limited reflow of molten metal, which results in an elevated deposition layer. Conversely, defects at the deposition end point stem from the abrupt termination of the arc, leading to insufficient reflow of molten metal and the formation of a slanted profile. Under conditions of high deposition current (250 A), the excessive arc force causes the molten pool to become deeper and wider. According to formula *Q* = *ηUI*/*v* (where *Q* is heat input, *η* is thermal efficiency of the arc, *U* is arc voltage, *I* is deposition current, and *v* is deposition velocity), a greater deposition current delivers more heat, resulting in inadequate heat dissipation and, consequently, more pronounced deposition layer defects. To address this issue, they employed a lower deposition current (200 A) and varied the travel speeds at the deposition start and end points (800 mm/min at the start, 400 mm/min at the end, with an intermediate speed of 600 mm/min), which makes the manufactured cup-shaped parts more continuous and smoother.

**Figure 3:**
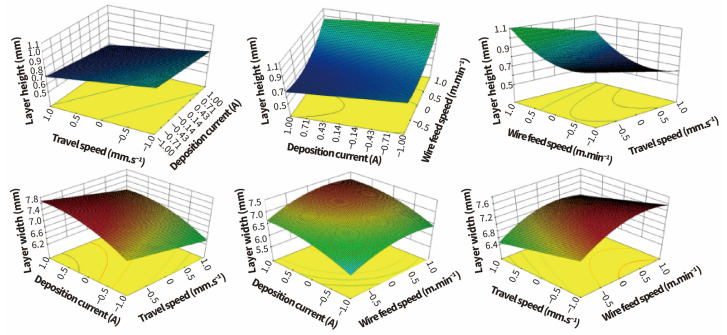
Response surface analysis of the interaction of plasma arc-based DED process parameters on the layer height and width of high-strength-steel additive manufacturing parts [65].

In order to prevent the accumulation of dimensional errors through layer-by-layer deposition, it is necessary to control the height and width of each deposition layer. Researchers have conducted extensive studies on the influence of process parameters (deposition current, arc voltage, travel speed, and wire feed speed) on deposition layer height and width. Particularly, GTA-based or PA-based DED processes generally adopt a bypass wire feeding system. The wire feed speed, arc voltage, and deposition current are decoupled, which can be freely adjusted within a certain range, making it more convenient to study the effects of each process parameter on deposition layer formation. Ouyang et al. [62] used the VPGTA-based DED technology to deposit 5356 aluminum alloy cylindrical wall-shaped structures on a 6061-T6 aluminum substrate. The results showed that deposition layer height significantly decreased with increasing travel speed (the travel speed increased from 50 mm/s to 100 mm/s) or deposition current (the deposition current increased from 70 A to 160 A), and the deposition layer width decreased with faster travel speed but increased with higher deposition current. When the travel speed is increasing, while keeping other parameters constant, the mass of molten metal per unit time remains unchanged. This leads to a reduction in the mass of the deposited metal per unit length along the bead, consequently decreasing the layer height. At the same time, the increase in travel speed leads to a reduction in the amount of heat received by the molten pool, resulting in a smaller molten pool size and a narrower deposition layer width. When only the deposition current is increased, the arc pressure on the molten pool surface and the impact of the molten droplets on the molten pool increases. This enhances the flowability of the molten pool, consequently reducing the layer height and broadens the layer width. Studies on PA-based DED technology have also yielded analogous findings [63]. However, the sensitivity of layer width to changes in travel speed differs from that of layer height. Notably, the sensitivity of layer height to changes in travel speed decreases as travel speed rises [64]. Liu et al. [65] studied the relationship between the forming dimensions of high-strength steel and the process parameters—deposition current, travel speed, and wire feed speed—in the PA-based DED process. They employed a response surface methodology to assess the impact of each parameter on the forming dimensions, as illustrated in Figure 3. The results indicate that the influence on layer height is ranked as follows: wire feed speed > travel speed > deposition current; for layer width, the ranking is as follows: deposition current > travel speed > wire feed speed.

It should be noted that the aforementioned conclusions may not be entirely applicable to GMA-based or CMT-based DED technology. As pointed out by Yildiz et al. [66], there is a specific matching relationship between deposition current and wire feed speed in CMT-based DED technology, and the arc voltage and deposition current can be adjusted by altering the wire feed speed of the CMT power source. In addition to the aforementioned methods that ensure the dimensional accuracy of the deposited layers, several real-time monitoring and control methods for the layer dimensions are also provided here. Some researchers have used structured light-based 3D scanners to quickly obtain three-dimensional information of the part surface, which can adapt to parts of different sizes [67]. Du et al. [68] constructed a real-time acquisition system for the morphology of the deposited layer during the deposition process using a light reduction filter, a color filter, and an industrial charge-coupled device (CCD) camera, and implemented the extraction of layer width and height based on the Halcon platform. Other researchers have used laser vision sensing systems to detect the contours during the deposition process, which can monitor in real-time whether the deposited layer height meets the design requirements [69], and passive vision sensing systems have also been used for real-time monitoring of the deposited layer dimensions in WA-DED [70]. Huang et al. [71] established a non-contact in situ 3D laser profilometry inspection (3D-LPI) system to automatically monitor visual surface defects, and with the support of the Point Cloud Library (PCL) and the Open-Source Computer Vision Library (OpenCV), they analyzed surface quality and defects through self-developed software. The aforementioned methods can all be used to inspect the deposition process. Zhang et al. [72] equipped with an arc deposition system with a 3D positioner and a torch rotation device, which can automatically calibrate the deposition height in real-time, eliminate cumulative errors, and improve forming accuracy. Xiong et al. [73] based on the monitoring of the nozzle to the top surface distance (NTSD) during GMAW deposition of multi-layer walls using a passive vision sensing system, designed an adaptive control system that compensates for NTSD deviations by adjusting the movement of the working plane and the travel speed of the next layer, maintaining a constant NTSD, thereby ensuring the constancy of the deposited layer height. Similarly, Franke et al. [74] established a wire segmentation detection algorithm based on TensorFlow machine learning and a spatter detection algorithm based on classic image processing techniques, where the wire detection algorithm can directly measure the distance from the nozzle to the workpiece and the horizontal position of the wire, while the spatter detection algorithm is used to identify welding instabilities. These studies can all provide references for better control of the height and width of each deposited layer.

In terms of controlling surface flatness or roughness in WA-DED processes, there is already a considerable amount of literature. Dinovitzer et al. [75] conducted research on the GTA-based DED technology and, after performing ANOVA analysis with the aid of Minitab 18 software, found that the deposition current had a significant effect on the top surface roughness of the single-layer beads. Within a certain range of deposition current (50–59 A), a peak value of surface roughness was observed. After reaching this peak, further increasing the deposition current would reduce the surface roughness. The surface flatness of the side surfaces of both multi-layer single-pass and multi-layer multi-pass parts correlated with layer height. An appropriate layer height helps to minimize the impact of step-like or wave-like appearances on surface flatness [76]. As mentioned above, layer height can be controlled by changing the wire feed speed and travel speed. Xiong et al. [77] studied the effects of GMA-DED process parameters on the surface quality of the side walls of multi-layer single-pass parts and found that at wire feed speed of 3.73 m/min and inter-layer temperature of 200 °C, travel speed exceeding 0.42 m/min could cause arc instability and decrease surface quality. If the travel speed and inter-layer temperature are kept constant at 0.3 m/min and 120 °C, increasing the wire feed speed leads to more deposited metal, raising layer height and promoting step-like appearances, which lowers surface quality. Therefore, lower travel speed and wire feed speed help improve surface quality. Research on high-speed welding based on theories such as backward-flowing-molten-metal effect, surface tension effect and other models indicate that excessively fast welding speeds can lead to molten pool instability and humped weld bead formation [78,79,80]. These studies offer insights into the molten pool behavior and deposited weld bead formation of WA-DED, where the weakly constrained molten pool exhibits greater instability than in traditional welding processes.

### 3.2. Heat Input

Research shows that appropriate heat input can help obtain more suitable molten pool sizes and cooling rates, which benefits the improvement of part surface quality and forming accuracy [81,82]. Obviously, the process parameters discussed in Section 3.1 are closely related to the heat input during deposition, and the complex interactions and constraints among these parameters may blur the focus of the study—the forming mechanism of the part. Therefore, it is essential to establish a direct link between heat input and deposition layer formation. Some researchers have used a comprehensive quantity related to heat input to replace the aforementioned parameters to study how to control the forming accuracy of the part. Sun et al. [83] employed linear energy density El (J mm^−1^) and volumetric energy density Ev (J mm^−3^) to replace the above parameters as process parameters, and found that a linear energy density of 400 J mm^−1^ was the critical threshold between the “formed” and the “unformed” additive weld bead formation phenomena.

**Figure 4:**
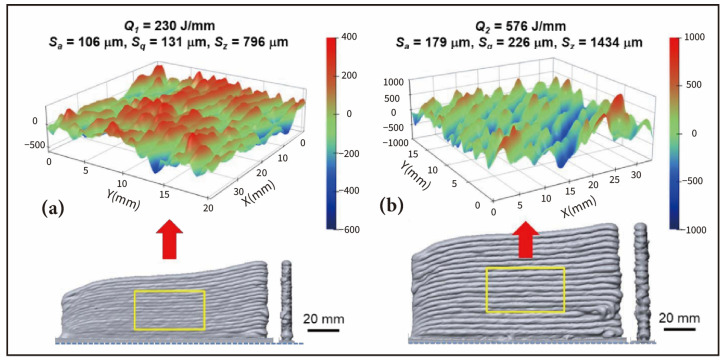
Surface flatness of a thin-walled parts deposited by WA-DED with heat inputs of (**a**) 230 J/mm and (**b**) 576 J/mm (the z-axis is measured in micrometers) [87].

The shape and the stability of the molten pool are decisive factors for the forming accuracy and surface quality of the parts [84]. Excessive heat input increases heat accumulation, leading to thermal stress and deformation of the parts [85], while insufficient heat input makes it difficult to stabilize the melting of the welding wire, affecting deposition efficiency. Research by Zhang et al. [82] found that when heat input was too high, surface flatness deteriorates. This could possibly be due to the reduced solidification rate of the molten pool, which made it more prone to overflow before solidification. This exacerbated the step-like and wave-like appearance, resulting in decreased surface quality. Real-time control of heat input during the deposition process is an important means to improve the forming accuracy of WA-DED parts [86]. However, the relationship between heat input and molten pool size is extremely complex, and further exploration is necessary to construct an accurate model of this relationship under the influence of multiple factors. This will provide key theoretical support for enhancing the shape control capability of WA-DED technology. Le et al. [87] investigated the relationship between heat input and surface roughness using GMA-DED by adjusting the heat input through variations in deposition current, arc voltage, and travel speed. They found that a heat input of 230 J/mm resulted in a higher surface finish compared to 576 J/mm, as illustrated in Figure 4. Furthermore, Zamiela et al. [88] integrated complex infrared (IR) thermal data during the deposition process with the Goldak double ellipsoidal heat flux to model the energy input into the deposited component. Subsequently, they utilized the thermal data-informed heat flux to create a thermal physics-informed model input (PIMI), which captured the internal thermal history. Finally, a regression convolutional neural network (CNN) was employed to capture the relationship between the three-dimensional thermal gradient and the resulting surface deformation, thereby predicting part deformation. This research fills the gap in fusing in situ thermal sensors, interpolating internal thermal histories, and extracting valuable thermal features for deformation detection in WA-DED, contributing to the production of parts with minimal deformation and low internal residual stress.

In terms of the correlation between heat input and residual stress, an increase in heat input typically results in an increase in residual stress levels, especially in the transverse direction of the deposited layers. This phenomenon is mainly due to the larger volume of material being heated, coupled with enhanced restrained shrinkage, which triggers higher tensile stresses during cooling, ultimately leading to part deformation [89]. Additionally, studies have indicated that microstructural alterations occur under different heat input conditions, encompassing changes in grain size and phase transformations. Specifically, higher heat input is linked to larger grain sizes, which also exerts an influence on the formation of residual stresses [90]. Furthermore, the microstructural evolution under varying heat input conditions, as well as the relationship between heat input and phase stability, will be comprehensively summarized in future works.

### 3.3. Welding Torch Orientation

WA-DED technology involves the layer-by-layer deposition of molten metal to manufacture parts. For components with overhanging features or inclined wall-shaped parts, traditional methods require support structures, which reduces the advantages of WA-DED in terms of material savings and may even lead to additional deformation and stress issues. Support-free deposition, if possible, would be well received in the industry. It may be achieved through adjusting the deposition angle, which includes modifying the orientation of either the substrate or the welding torch. There is relatively little research on the former due to the significant difficulty and enormous additional costs associated especially with the large-scale part deposition, at which WA-DED excels.

**Figure 5:**
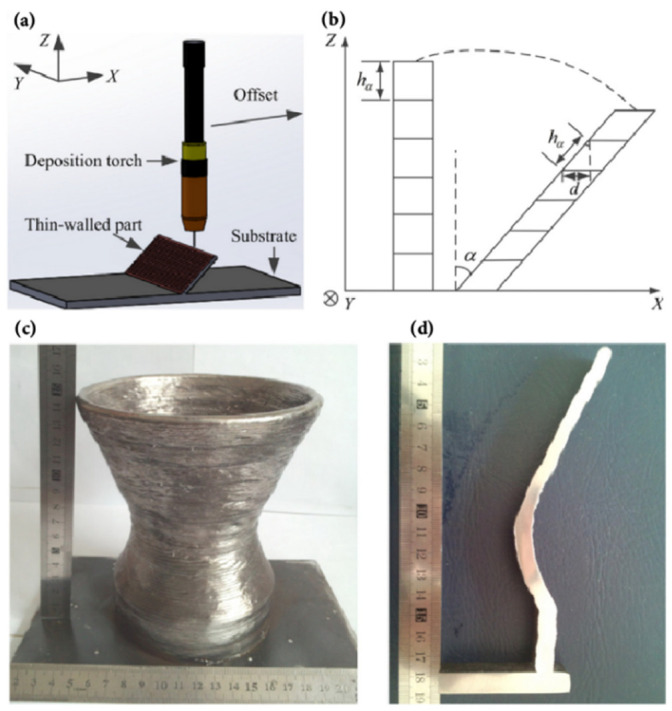
Small-angle-inclined parts manufactured by GMA-DED: (**a**) schematic diagram of the deposition process and welding torch orientation; (**b**) schematic diagram of the inclination angle *α*; (**c**) a deposited cup-shaped part; (**d**) cross-section of the deposited part [91].

For parts with a small inclination angle, the torch can be positioned vertically to the substrate. Xiong et al. [91] manufactured inclined parts with a small tilt angle based on GMA-DED and studied the relationship between deposition speed (equal to travel speed of the welding torch) and inclination angle *α*. The orientation of the torch is shown in Figure 5a, while the definition of inclination angle *α* is illustrated in Figure 5b. An additively deposited part and its cross-section are shown in Figure 5c and Figure 5d, respectively. When other parameters remain unchanged, an increase in deposition speed results in a decrease in layer height *h_α_*, and it can be observed in Figure 5b that the inclination angle *α* increased at the same time.

**Figure 6:**
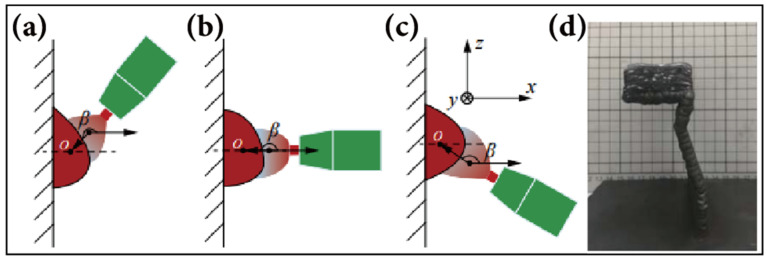
Deposition of the overhanging parts: (**a**–**c**) schematic diagram of the welding torch orientation; (**d**) an overhanging part manufactured via WA-DED [92].

When depositing parts with a large inclination angle or overhanging structures, the force of gravity tends to pull the metal fluid of the molten pool downward, which may lead to a deterioration of forming accuracy and surface quality. To address the aforementioned issues, Wang et al. [92] made adjustments to the welding torch angle β. As illustrated in Figure 6a–c, when β exceeds 180°, both the arc force and gravity pull towards the negative z-direction, leading to a greater accumulation of solidified metal in the lower half of the deposited layer compared to the upper half. When β equals 180°, the arc force is perpendicular to the z-axis, and the molten material is solely influenced by gravity, yet the lower section still exhibits a higher concentration of solidified metal. However, when β falls between 90° and 180°, the component of the arc force along the positive z-direction counterbalances the gravity pulling in the negative z-direction, significantly minimizing the dripping of molten material. Ultimately, by fine-tuning β to 135°, they successfully deposited parts featuring overhanging structures. Kazanas et al. [93] successfully deposited thin-walled and complex structural parts (square section part, overhanging part and semicircle part) with inclination angles of 0°, 15°, 30°, 45°, 60°, and 90° by precisely controlling the angle between the torch and the substrate using CMT-based DED technology.

In the aforementioned study, the deposition forming accuracy and surface quality of parts with large inclination angles or overhanging structures are not very good. Researching the droplet behavior and the fluid flow of the molten pool at various torch inclination angles helps to better address the issue of molten pool flowing downward. Additionally, applying energy fields to alter the force conditions on the droplets and molten pool may prevent the molten pool from sagging, thereby controlling forming accuracy and surface quality.

### 3.4. Control of Droplet Behavior

A molten droplet is the basic unit of mass transfer in the WA-DED process. The force conditions (magnitude and direction) determine the size and the transfer frequency of the molten droplet. The droplet size and transfer frequency can directly affect the metal transfer mode and the impact force on the molten pool, thereby influencing the morphology and stability of the molten pool. Therefore, effective control of droplet size and transfer frequency can directly improve the forming accuracy and surface quality of WA-DED parts [94]. In the realm of WA-DED technology, two primary modes of droplet transfer are identifiable: free transfer and bridging (or short-circuit) transfer. In free transfer, droplets emerge at the tip of the welding wire, growing steadily under the action of surface tension until they reach a weight that surpasses the supportive capacity of the surface tension, ultimately detaching and plunging into the molten pool. Spray transfer, a specific type of free transfer, involves the rapid, high-frequency transition of minute spherical droplets from the welding wire tip to the molten pool. As arc power intensifies, so does the impact force on the molten pool, which, if excessive, may induce deposited layer collapse. In bridging transfer mode, the welding wire tip is in intimate proximity to, or even in direct contact with, the molten pool surface, fostering the formation of a liquid bridge—liquid metal linking the wire tip and the molten pool. Compared to free transfer, bridging transfer exhibits smoother droplet transfer dynamics and prolonged droplet–molten pool contact time, conducive to achieving a more uniform and smoother deposited layer [95,96]. Luo et al. [96] proposed a calculation method for the average droplet size under spray transfer mode for pulsed GMA-DED, pointing out that when the arc power is too high, the electromagnetic contraction force and plasma flow force increase, which restricts the growth of droplet size and increases the transfer frequency, raising the heat input and droplet impact force, making the deposition layer easy to collapse. Lü et al. [97] found that the increase in current density and Lorentz force would enhance the axial thrust of the arc, thereby accelerating the droplet transfer. Cao [98] studied the momentum of molten droplets undergoing oscillatory motion due to electromagnetic forces, gravity, etc., noting that smaller molten droplet oscillation momentum helps stabilize the molten pool, which can serve as a characteristic parameter for assessing the surface quality of parts. Additionally, in the PA-based DED process, the plasma arc exerts a strong plasma flow force on the molten droplets, resulting in good directionality of the droplets during deposition. Therefore, PA-based DED offers unique advantages in controlling the droplet transfer path [77].

**Figure 7:**
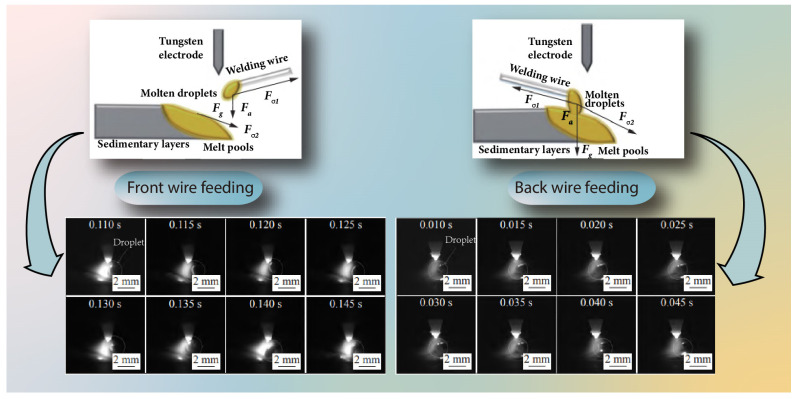
Transition of molten droplets between front wire feeding and back wire feeding during GTA-based DED [99].

Generally speaking, free molten droplet transfer has a significant impact on the molten pool and can easily induce instability in the deposition layer formation [95]. Li et al. [99] achieved a DED process for bridging transfer (commonly referred to as short circuiting transfer in welding) based on the step feeding dual-pulse TIG arc method, resulting in good forming accuracy. Moreover, it was found that the forming accuracy of the front wire feeding method is lower than that of the back wire feeding method, as shown in Figure 7. When using the front wire feeding method, the vertical distance from the melting tip of the welding wire to the molten pool surface is relatively large. This distance leads to an excessively large droplet size during the bridging transfer process, which has a significant impact on the molten pool, resulting in an unstable metal transfer process and degraded forming accuracy.

**Figure 8:**
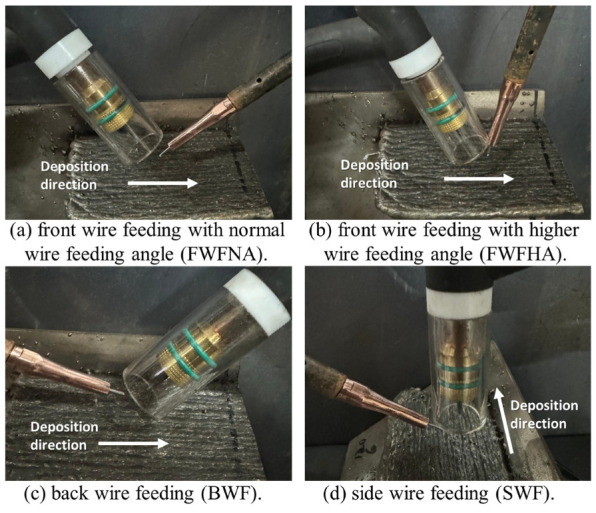
Various wire feed setups for bypass wire feeding GTA-based DED and the bead forming of the two types of front wire feeding angles.

**Figure 9:**
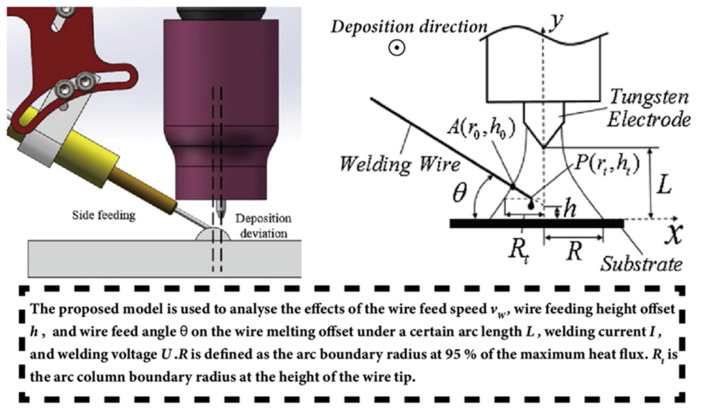
Schematic of deposition deviation of side feeding GTA-based DED [101].

In WA-DED with bypass wire feeding method, the direction of forces on the molten droplets may not be along the welding wire axis, and the heat is not symmetrical. Therefore, the molten droplet transfer process may be unstable, requiring attention to the welding wire melting rate and the droplet landing position. It has been found that using the front wire feeding with a normal wire feed angle—where the welding wire is positioned in front of the welding torch at an angle of 18 degrees to the substrate, as illustrated in Figure 8a—results in better dimension accuracy than using a front wire feeding with a higher wire feed angle (85 degrees to the substrate, as shown in Figure 8b) [100]. Figure 8c,d show the back wire feeding and the side wire feeding methods, respectively. Wang et al. [101] considered the deviation of the molten droplet’s landing point into the molten pool relative to the tungsten electrode axis when using the GTA side feeding method, as depicted in Figure 9. This means that the transfer of mass and heat is not symmetrical along the arc axis and may even be completely distributed to one side, leading to deposition deviation. A melting model for side feeding GTA-based directed energy deposition was established, proposing the control of deposition parameters to optimize the welding wire melting offset of the side feeding method and eliminate deposition deviation, thereby improving the forming accuracy.

### 3.5. Inter-Layer Temperature and Preheating

One of the primary factors contributing to the instability of the melt pool is excessive heat accumulation, which often requires heat dissipation from the deposited layers to achieve an inter-layer temperature suitable for continued deposition. In WA-DED, the inter-layer temperature refers to the temperature of the previous deposited layer just before a new one is deposited [102]. At lower inter-layer temperatures, the melt pool can solidify and form more quickly. Conversely, at excessively high inter-layer temperatures, the melt pool cannot solidify in time, resulting in a “mixed layer” phenomenon. This phenomenon refers to an irregular appearance where adjacent layers are blended together [60,86]. Therefore, the inter-layer temperature has a significant impact on the forming accuracy and surface quality of the additive manufactured parts [77].

The inter-layer temperature is directly related to the heat input and can be controlled by adjusting parameters such as deposition current, arc voltage, travel speed, and wire feed speed. In the context of continuous multi-layer deposition for cup-shaped parts, Zhao et al. [103] classified different deposition stages based on changes in cooling conditions, developed an inter-layer temperature prediction algorithm. Additionally, they designed an adaptive model that can adjust process parameters in real-time according to the predicted inter-layer temperature. This enabled a consistent layer dimension in continuous deposition processes and was validated on a large shell-shaped part consisting of 753 layers. It is important to note that while excessive adjustment of process parameters to reduce heat input can lower inter-layer temperatures and heat accumulation, it will inevitably result in a decrease in deposition efficiency.

Some researchers have proposed preheating the substrate to reduce the temperature gradient, decrease the generation of thermal stresses, and thus prevent deformation [104]. The required preheating temperature varies with different substrate materials. For example, Ouyang et al. [62] preheated a 6061-T6 aluminum substrate to 118 °C when depositing 5356 aluminum alloy to improve thermal deformation and reduce the temperature gradient. Cai et al. [105] preheated the substrate to 450 °C when depositing a TiAl-based alloy with 50% aluminum content (atomic fraction) to enhance the performance of the part. Xiong et al. [106] established a three-dimensional finite element model to numerically simulate the thermal behavior during deposition with H08Mn2Si welding wire at various preheating temperatures. They indicated that preheating temperatures between 400 °C and 600 °C are beneficial for reducing thermal stress and preventing part deformation. It is worth noting that the preheating temperature should not be set to be excessively high. Shi et al. [107] observed that when depositing with ER2319 welding wire on a 6060 aluminum alloy substrate, preheating temperature exceeding 120 °C can lead to significant thermal accumulation, resulting in a marked increase in the width and depth of additive weld beads.

Preheating the substrate before deposition can reduce the temperature gradient, while after deposition begins, cooling is often required to lower the temperature gradient and expedite the solidification of the melt pool. Extending the cooling time between layers, although it does not make the melt pool solidify faster, can achieve lower inter-layer temperatures and temperature gradients (except for the current deposited layer). Under air-cooling conditions, three primary methods exist for controlling interlayer temperatures: a preset interlayer cooling time [108,109], real-time temperature monitoring during deposition process, and predictive modeling to forecast interlayer cooling times. However, the first approach, utilizing a fixed interlayer cooling time, cannot maintain a consistent temperature throughout the deposition process due to the escalating temperature gradient as additional layers are added [110]. Consequently, to ensure a stable interlayer temperature, it is imperative to employ either real-time temperature monitoring during the deposition process or predictive modeling to forecast the interlayer cooling time for each subsequent layer.

**Figure 10:**
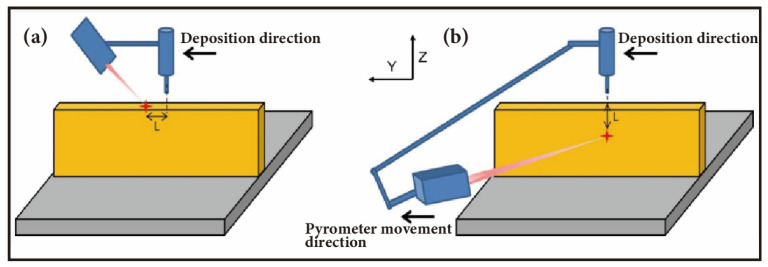
Interlayer temperature detection position strategy: (**a**) the upper pyrometer strategy and (**b**) the sideward pyrometer strategy [117].

For the second method, real-time temperature monitoring during the deposition process, some researchers use pyrometers, surface thermometers, and infrared thermometers for interlayer temperature measurement. For instance, Jiang et al. [111] used a pyrometer to measure the temperature at the center of the top surface of the last deposited layer every 2 s and initiated the deposition of the next layer once the temperature cooled to the target value in the air. They found that the optimal interlayer temperature range for depositing with Hishiko OMH-1 welding wire on an H13 steel substrate is between 150 °C and 200 °C. Ogino et al. [112] employed a surface thermometer to measure the temperature at the start of the next layer deposition, ensuring a constant surface temperature of 573 K. This can prevent the deposited layers from reliquefying due to excessive temperature and collapsing under gravity. Some researchers use thermocouples on the substrate for temperature detection, setting reference points on the substrate to monitor temperature as a proxy for interlayer temperature [113]. While this method is convenient, as the number of deposited layers increases, the measurement points become distant from the molten pool, leading to potential errors in measurement [114]. Some researchers have also employed various temperature monitoring systems to detect temperatures in real-time during the deposition process, which can also provide a reference for interlayer temperature detection. For instance, Liu et al. [115] used a pyrometer and an infrared camera as a temperature detection system to monitor the temperature distribution of the melt pool during the deposition process. Tan Hua and his team established a temperature measurement system for laser rapid forming using a two-color infrared pyrometer, which is capable of real-time tracking and spot measurement of the temperature in the melting zone [116]. Regarding the positioning for interlayer temperature measurement, Jorge et al. [117] identified two strategies: the upper pyrometer strategy and the sideward pyrometer strategy. As shown in Figure 10, the upper pyrometer strategy involves measuring the interlayer temperature at a specific point on the top surface of the last deposited layer, while the sideward pyrometer strategy measures at a point on the side of the deposited layer below the molten pool. A comparative analysis of these two strategies revealed that the upper pyrometer strategy is more suitable for long wall lengths and wall extremities, whereas the sideward strategy has low measurement sensitivity and is impractical for the initial layers. Therefore, both methods have their limitations. The third method involves using models to predict interlayer cooling times, as demonstrated by Montevecchi et al. [118], who conducted finite element analysis (FEA) of the DED process to solve the heat transfer equation and predict dynamic interlayer cooling times, achieving a constant interlayer temperature. This method eliminates the need for thermocouples or temperature sensors, avoiding measurement errors. However, its FEA model is based on substrate thermocouple measurements, and research shows that using substrate temperature as a proxy for interlayer temperature can introduce errors.

**Figure 11:**
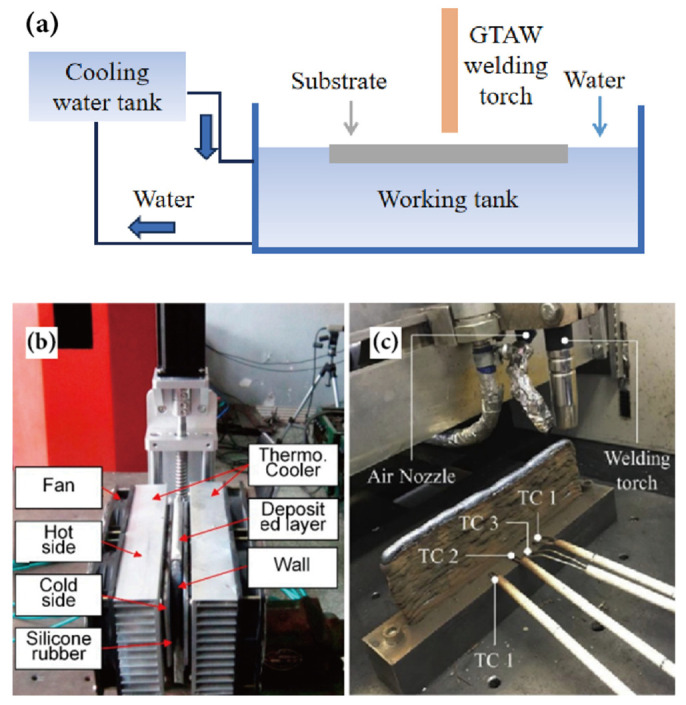
Various external cooling devices for WA-DED: (**a**) a water-bath platform; (**b**) a semiconductor cooling system [122]; (**c**) an air jet device [123].

However, under conventional conditions, the primary cooling mechanism for additively manufactured metal parts is through heat conduction only within the metal perpendicular to the substrate direction [119], while the heat exchange between the surfaces of parts and the air is very low. Achieving the desired lower inter-layer temperature under air cooling conditions takes a long time, and the cooling time of the melt pool is slow, which can easily reduce deposition efficiency. When it comes to air-cooling, the duration required for various deposited layers to reach their target temperatures differs. Take the GMA-DED deposition process as an example: under the process parameters of a deposition current of 120 A, an arc voltage of 20 V, and a travel speed of 4 mm/s, a wall measuring 180 mm in length was deposited with 20 layers. A temperature gun was utilized to measure the temperature at the center of the top surface of the final deposited layer. It was observed that the second layer needed 10 s to cool to 50 °C, whereas the 16th layer required 14 s [111]. Furthermore, distinct deposition techniques result in varying air-cooling durations. For instance, in CMT-based WA-DED technology, with an average deposition current of 255 A, an average arc voltage of 16.5 V, a travel speed of 0.9 m/min, and a wire feed speed of 8 m/min, it takes approximately 200 s to cool down to 200 °C [120]. Notably, environmental temperature also plays a significant role in influencing air-cooling time. Artificially enhancing the cooling process is an important strategy for continuously depositing large-scale parts and achieving good formation. Adding external cooling equipment for forced cooling can reduce the melt pool temperature and the temperature gradient of the already deposited parts. Duan et al. [121] added a water tank below the substrate for heat dissipation. They compared this method with air-cooled deposition for the manufacturing of a single-pass 10-layer wall and found that the substrate deformation under the water bath condition was smaller, and the standard deviation of the heights of the wall measured at 10 mm intervals was reduced by 16%. Also, the deposited layers’ collapse phenomenon at the arc termination end was improved to a certain extent. Li et al. [122] introduced an active cooling system composed of semiconductor refrigeration and heat dissipation fins into WA-DED, which forced the cooling of additively manufactured parts and improved the forming quality. However, this system requires continuous adjustment of its position according to the current height and shape of the parts during depositing. Hackenhaar et al. [123] effectively controlled the inter-layer temperature using a system of air jetting, but this equipment may lead to a reduction in the protective effect of shielding gas and oxidation of the WA-DED parts. Figure 11 shows relevant images of the various external cooling devices mentioned above. The utilization of external cooling equipment can significantly enhance the cooling rate, but there remains considerable scope for advancing the adaptability and flexibility of such devices.

### 3.6. Substrate Design

**Figure 12:**
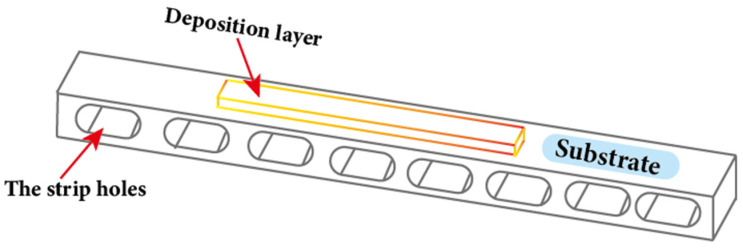
Schematic diagram of long strip holes on the substrate for DED additive manufacturing.

The design of the substrate is equally critical as the process parameters mentioned above for WA-DED technology, and current research primarily focuses on the design aspects of substrate thickness and structure. Some researchers have found that increasing the thickness of the substrate not only improves stiffness, increases constraints, and reduces deformation, but also strengthens heat dissipation, thereby reducing the heat accumulation. This is advantageous for enhancing forming accuracy, and is beneficial to the deposition of large-scale, monolithic parts [124]. However, the thickness of the substrate is not the thicker the better. As the thickness of the substrate increases, the heat dissipation can become too strong, making it challenging to achieve a wider molten pool, which in turn limits the width of the deposited layer [107]. Researchers introduced long strip holes in the substrate, as shown in Figure 12, and found that residual deformation could be reduced by 60% [125]. These long strip holes change the heat dissipation characteristics and rigidity of the substrate, altering the temperature distribution. This reduces the temperature gradient in both the high-temperature region of the substrate and the deposition layers, thus reducing thermal stresses and improving the dimensional accuracy of the additively manufactured structures. In order to meet the requirements for depositing large-scale, integral parts, in addition to adjusting the substrate thickness and structure, factors including material and substrate preheating should also be considered. It is highly recommended to develop a comprehensive substrate design system that takes into account both heat dissipation and constraint conditions.

Processing parameters are one of the fundamental elements that determine the microstructures of additively manufactured metals [126]. Here are the microstructural traits of parts fabricated through various WA-DED techniques under specific processing conditions. Utilizing GMA-DED with a deposition current of 320 A, an arc voltage of 28 V, and a wire feed speed of 104 mm/s to deposit ER70S-6 low-carbon steel revealed a range of microstructural features in the heat-affected zone, including grain coarsening, the emergence of non-equilibrium phases (acicular ferrite AF and bainite B) adjacent to the melt pool boundaries, and localized brittle zones attributed to martensite-austenite phases. These were consequences of microstructural inhomogeneity stemming from intricate thermal cycling [127]. In contrast, depositing 2209 duplex stainless steel (DSS) with a deposition current of 155 A, an arc voltage of 25 V, a travel speed of 12 mm/s, and a WFS of 147 mm/s showed that the first layer contained a higher ferrite content (35%) compared to the intermediate layers (ranging from 25% to 30%) [128]. When depositing Ti-6Al-4V alloy using TIG-based arc DED, with deposition currents of 180 A and 140 A, and WFS for the final layer between 1400 and 2600 mm/min, at table speeds of either 0.25 m/min or 0.3 m/min, the microstructures were broadly similar, characterized by a coarse Widmanstätten structure with acicular lamellae in the bottom region and a finer acicular structure in the top. Notably, cooling rate plays a pivotal role in determining microstructure; sufficiently rapid cooling (above 20 K/s) can induce the formation of martensitic structures [129]. Future works will delve deeper into the effects of process parameters in WA-DED technology on microstructural attributes such as grain size, phase distribution, and porosity.

### 3.7. Surface Tension and the Related Marangoni Effects

Surface tension and the related Marangoni effects play a significant role in molten pool dynamics [130,131]. The distribution of surface tension on the interface of molten pool and the arc atmosphere is related to the temperature dependence of the surface tension coefficient, i.e., the surface tension temperature gradient ∂γ/∂*T* [132]. And this distribution has a marked effect on weld depth and profiles because it can influence the direction of circulatory flow in the weld pool [133]. For common weld pools under the heating effect of the arc, the center of the weld pool surface has a high temperature, while the edge has a lower temperature. With a positive surface tension temperature gradient (∂γ/∂*T* > 0), the surface tension in center of the weld pool surface is greater than that in the edge area. The melting metal near the surface flows from the outside towards the center and then down towards the bottom of the weld pool, which brings the arc’s heat to the bottom, increasing the penetration depth [134], while when the surface tension temperature gradient is negative (∂γ/∂*T* < 0), the weld pool penetration becomes shallower while the width increases. The flow phenomenon driven by the distribution of surface tension mentioned above is the well-known Marangoni effect.

However, for the WA-DED molten pool, the situation may differ. Unlike in welding processes where the base metal is on both sides of the molten pool, the sides of the WA-DED molten pool (in multi-layer single-pass) or at least one side (in multi-layer multi-pass) often lack solid metal [135]. Consequently, heat dissipation to the sides is lower, and the temperature difference between the center and the edges of the molten pool surface is limited. Furthermore, in the WA-DED process, the current is typically not higher than 200 A, and more heat is desired to be used for melting the wire rather than maintaining the molten pool [136]. Under these circumstances, the molten pool is relatively small, the time of the molten metal above the liquidus temperature is reduced. It can be inferred that the Marangoni effect’s role in the WA-DED molten pool is relatively limited [137]. But on the other hand, the influence of surface tension on the cross-section profile of the bead is highlighted [122]. Surface tension itself may determine the degree to which the molten pool “bulges” on the base metal or the preceding deposited surface, especially because of lacking confinement from base metal on both sides [138]. A capillary-gravity model is often used to analyze the contour of the bead, in which the difference in capillary pressure between the bulging surface of the molten pool and the original surface prior to the deposition is balanced against the force of gravity [138,139]. The capillary pressure *P*_c_ is caused by surface tension and is calculated according to Laplace’s law, which follows the formula:

(1) $$P_{c}=\gamma \;(\frac{1}{r_{x}}+\frac{1}{r_{y}})$$

in which γ is the surface tension coefficient, which is often assumed to be constant over the melt surface. And *r_x_* and *r_y_* are the radii of curvature at *y* and *x* planes, respectively.

A higher surface tension can maintain a greater degree of central bulging in the molten pool, resulting in a larger layer height and a relatively narrower layer width. At higher deposition speeds or when depositing uphill on a sloping surface, surface tension plays a role in promoting the formation of humping defects [140].

In welding processes, it is often desirable to increase penetration and reduce the heat-affected zone, with the common method: the addition of surface-active elements such as S, O, Se [141]. However, in WA-DED, it is usually desirable to decrease penetration, as greater penetration can increase the remelting proportion of previously deposited layers, potentially leading to coarser grain size. When considering the surface-forming quality of additive parts, as described in Section 3.1, sometimes, a wider layer width with a lower layer height is preferred, thus welcoming a negative surface tension temperature gradient. The surface tension of binary solutions of iron–sulfur and iron–oxygen have been extensively studied from the 1950s to the 1980s and are well-known to welding scholars [142]. Near the melting point, the surface tension of low-sulfur carbon steel and stainless steel is between 1.7 to 2.0 N/m. Moreover, for most of the temperature range above the liquidus line, the temperature gradient is often negative [143,144]. Therefore, the addition of elements such as sulfur and oxygen can reduce the surface tension over the entire molten pool surface, which is conducive to reducing layer height; however, on the other hand, it may lead to a positive surface tension temperature gradient, causing inward circulation that hinders the spread of the molten pool [145,146]. Additionally, turbulence in the molten pool under the addition of surface-active elements has also been observed, even though it has been traditionally believed that the flow within the molten pool is predominantly laminar. In summary, the optimal ratio of surface-active elements to be added still needs to be investigated.

## 4. Deposition Path Optimization

**Figure 13:**
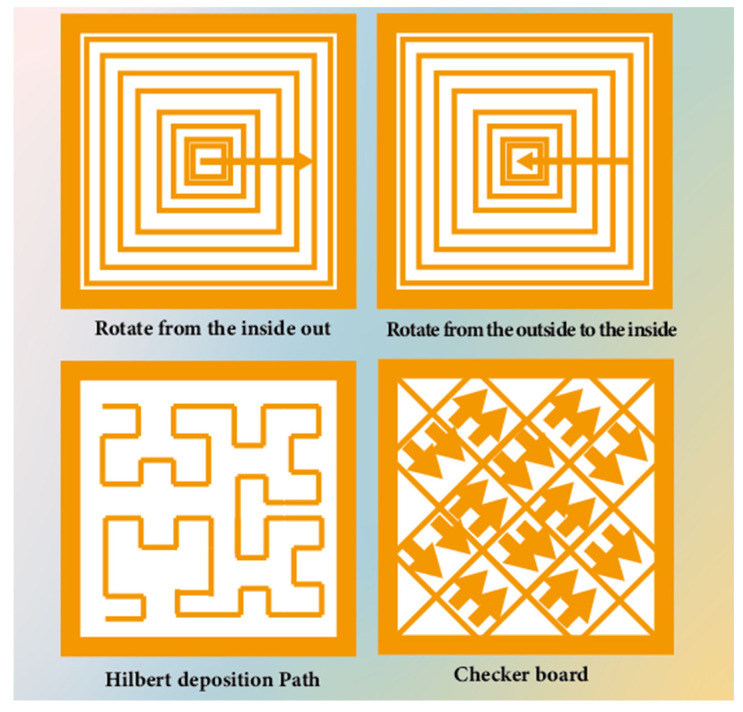
Schematic diagram of typical deposition paths.

**Figure 14:**
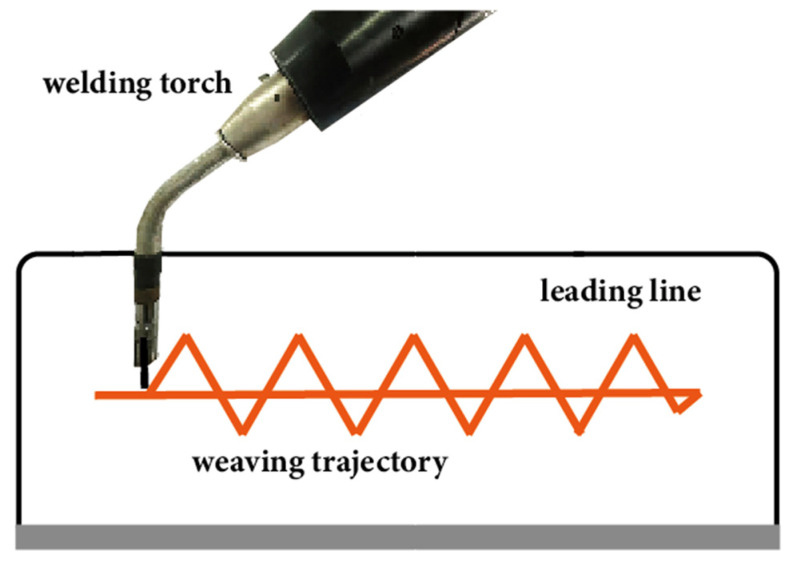
Schematic of deposition with torch oscillating (along the weaving trajectory).

Utilizing WA-DED technology, variations in the deposition path arrangement can lead to changes in the internal temperature distribution and stress condition of the additively manufactured parts, resulting in different residual stress and deformation [147,148]. For simple geometric shapes, each deposition layer can be segmented into multiple parallel and overlapping weld beads, which are commonly deposited in sequence. During actual manufacturing, the deposition paths within each layer can vary depending on the specific conditions, including patterns such as rotating from the inside out, rotating from the outside to the center, or checkerboard deposition [149,150], as shown in Figure 13. In Figure 13, the spiral patterns (rotating inwardly from the center and outwardly from the perimeter) contribute to complex thermal cycling. The outwardly-to-inwardly rotating pattern exhibits higher residual stresses due to the accumulation of heat towards the center, whereas the inwardly-to-outwardly rotating pattern distributes heat outwardly, resulting in relatively lower residual stresses. Furthermore, the residual stresses associated with the Hilbert deposition path are greater than those of the spiral patterns. The technique of the welding torch periodically oscillating in a certain regularity while advancing is commonly found in welding and WA-DED processes, making each deposited weld bead to widen, thereby reducing the number of passes per layer, as shown in Figure 14 [151]. When the welding torch oscillates, the width of the molten pool expands and the height of the deposition layer sometimes decreases, resulting in a smoother top surface. Research by Yang et al. [152] found that as heat input increases, an appropriate arc oscillating width can improve the overlap quality of adjacent additive weld beads.

The deposition paths of adjacent layers are primarily characterized by the unidirectional (same direction) deposition, the counter-directional (reverse direction) deposition, or the perpendicular deposition (the angle between depositions in adjacent layers is 90°). In multi-layer additive manufacturing processes, deposition in reverse direction is frequently applied. This method helps to reduce distortion and improve the overall quality of the manufactured part by minimizing the heat accumulation and thermal stress that can occur in the same-direction deposition. Zhao et al. [110] found that when using GMA-DED for depositing multi-layer single-pass walls, the temperature gradient for unidirectional deposition is larger than that for counter-directional deposition. Huang et al. [153] discovered that the stress distribution with deposition in reverse directions is more uniform than deposition in the same direction, and that parts produced by reverse-directional deposition exhibit 25% less deformation. Other studies have shown that the perpendicular deposition improves the microstructure and tensile properties of the parts, reducing porosity [154].

**Figure 15:**
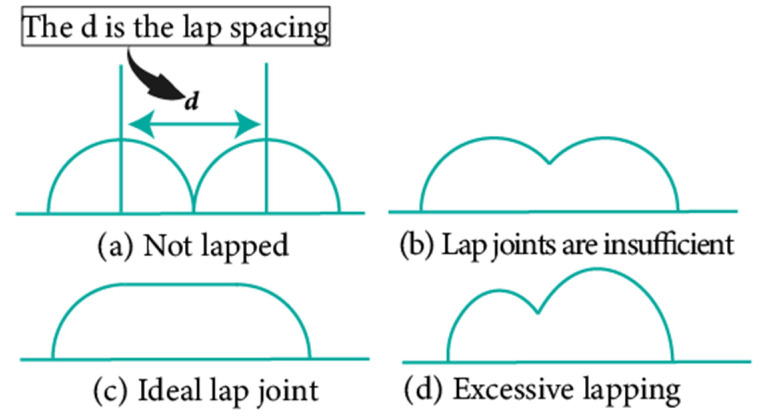
Four types of weld bead overlap spacing conditions.

In the design of deposition paths, the interbead spacing, i.e., the spacing between the centers of adjacent additive weld beads, particularly when depositing multi-pass parts via WA-DED, whether single-layer or multi-layer, is a critical parameter that requires meticulous consideration. An optimal interbead spacing can achieve better surface quality and form accuracy of the additively manufactured parts. The overlap effects of additive weld beads can be categorized into four types, as shown in Figure 15. Intuitively, insufficient overlap and no overlap can lead to reduced surface smoothness, while excessive overlap decreases deposition efficiency. Most researchers have established weld bead overlapping models to obtain optimal interbead spacing, including a relatively simple flat-top overlapping model (FOM), which assumes a flat cross-section connecting the cross-sectional profiles of adjacent deposited weld beads [155]. Ye et al. [156] used a dual-wire welding robot system for the deposition of high-nitrogen steel–stainless steel heterogeneous materials, employing a simplified arc cross-section model to describe the cross-section of the deposited weld bead and predicted the optimal weld bead spacing. Cao et al. [157] fitted the interface profile of GMA-DED deposited weld beads using a sine function and theoretically calculated the optimal overlap coefficient to be 63.66%. However, the actual morphology of the overlapping area often deviates from a flat plane, instead forming an undulating topography characterized by varying heights, akin to a landscape with ridges and valleys. Fang et al. [158] improved upon the FOM weld bead overlap model by using a developed parabolic function model to fit the profile of the overlapping area and found that the optimal interbead spacing for CMT-based DED technology is 0.715 times the weld bead width. Ding et al. [159] further developed the tangent overlapping model (TOM) by building upon the foundation overlap model (FOM), employing it to fit overlapping regions. Additionally, they introduced a method for calculating the critical center distance, also known as optimal interbead spacing, to delineate between stable and unstable overlaps. Their calculations revealed a critical center distance *d*, of 0.738 w (w denoting the width of the weld bead). When compared to FOM, the TOM demonstrated superior predictive capability in enhancing welding overlap quality.

The above study used the strategy of equidistant moving torch for deposition. However, surface tension leads to an additional pressure *P*_a_ on the curved liquid surface at the molten pool’s boundary, pushing the molten pool to move towards the preceding deposited beads, causing weld bead offset [160]. This additional pressure is numerically equal to twice the surface tension coefficient divided by the radius of curvature of the curved liquid surface. And the direction of this pressure points towards the center of curvature of the curved liquid surface. When one side of the molten pool has a preceding deposited bead, the radius of curvature on that side is smaller, resulting in a greater additional pressure, which causes the molten pool movement and weld bead offset mentioned above. Therefore, the relatively simple weld bead spacing model mentioned in the previous paragraph needs to be improved. Li [160] established an overlap model that takes into account the phenomenon of overlapping weld bead offset. At the same time, a non-equidistant movement strategy of the welding torch was adopted. This model predicted the overlap profile more accurately. Adjusting the process settings based on the predictions of this model significantly improved the surface flatness of parts manufactured via WA-DED with a six-layer, six-pass deposition process compared to the conventional overlap models. The four main types of models describing the phenomenon of additive weld bead overlap are listed in Table 2.

**Figure 16:**
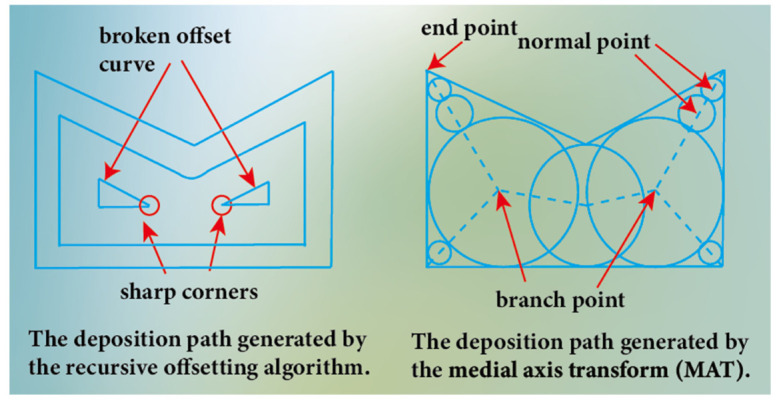
Comparison of the effect of deposition paths generated by recursively offsetting algorithms and medial axis transformation (MAT).

In order to address the issue of determining the optimal starting position for the deposition of complex parts, Lockett et al. [161] considered various factors including substrate waste mass, the mass of deposition material, and the number of deposition operations, deposition complexity and symmetry. They employed a multi-attribute decision matrix to evaluate the pros and cons of different deposition starting positions. Welding robots are often utilized in WA-DED technology. Conventional robot programming with a teach pendant affiliated to the robot is generally very time-consuming, especially for complex paths. Mehnen et al. [162] developed a path generation program suitable for Fanuc robots and a user interface for parameter settings, simplifying the robotic programming challenges of complex path deposition. Targeting the repair of damaged parts, Zheng et al. [163] developed a welding torch posture solving algorithm based on three-dimensional point cloud data of the parts’ surface, which can dynamically adjust the welding torch posture according to the local morphology of the damaged area, accommodating complex spatial constraints and ensuring repair quality. When performing overall path planning, the deposition paths generated by the recursively offsetting algorithms may exhibit sharp corners and discontinuities. Kao et al. [164] proposed a method based on medial axis transformation (MAT) to generate deposition paths to address these issues. The medial axis is used to describe geometries featuring a medial axis. For instance, in two-dimensional space, the medial axis is defined as the center of the maximum local circle within the geometry. While generating the deposition path based on MAT, the process fills from the inside out to produce a part without gaps. This is shown in Figure 16. However, this method is not applicable to parts of arbitrary geometries. In this regard, Ding et al. [165] innovated this method to generate gap-free deposition paths for any geometric shape, enhancing its universality. It should be noted that since the deposition using this method will fill the boundary with excessive material, post-processing of the part is required. Currently, researchers have applied overall path planning to actual manufacturing processes. For example, Li et al. [166] utilized the open-source 3D printing software Ultimaker Cura (more information can be found in https://ultimaker.com/software/ultimaker-cura/ (accessed on 14 November 2024)) as a plugin, importing the battery pack into Grasshopper for digital linkage slicing and printing path planning. By adopting an “adaptive member addition” algorithm to filter out the skeletal body with the lowest material occupancy rate (0.427) and using OpenCV image processing technology to prevent overhang printing, among a series of optimizations for the deposition process, they managed to control the design and manufacturing iteration cycle of a car chassis within one week, reducing the development cycle by 120 times.

**Figure 17:**
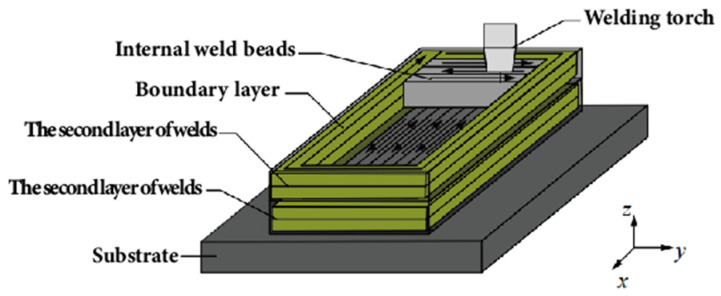
Schematic diagram of the deposition strategy of pre-depositing the boundaries and the perpendicular depositing [167].

**Figure 18:**
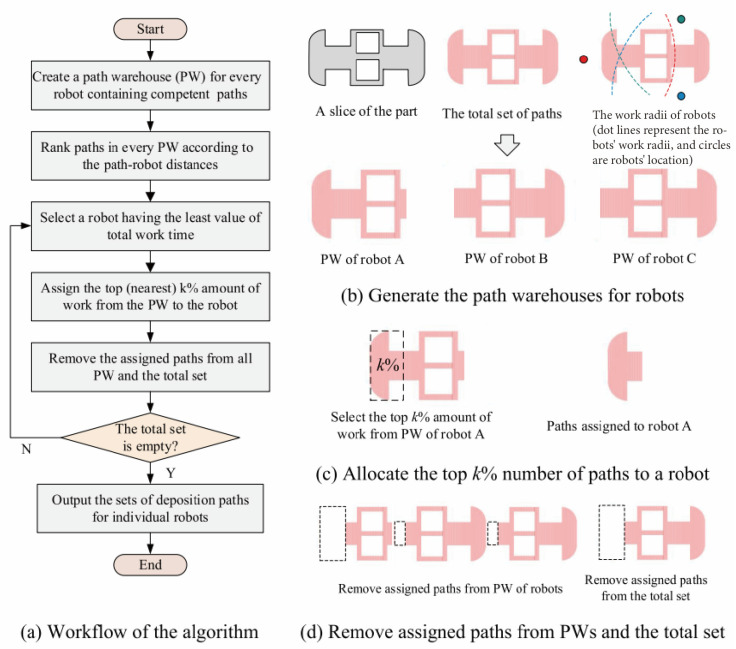
Algorithm for assigning deposition paths for multiple additive robots [168].

For multi-layer multi-pass thick-walled parts, Yang et al. [167] proposed a strategy of pre-depositing the boundaries of each layer to enhance constraints and improve edge collapse. Moreover, they employed the perpendicular deposition method, as discussed in the second paragraph of this chapter, for the odd and even layers to address the height discrepancy phenomenon on the surface of the deposited layers, as illustrated in Figure 17. To address the path allocation and scheduling issues during collaborative deposition with multiple robots, Li et al. [168] proposed a “top k%” path allocation and scheduling algorithm strategy. The core idea is to allocate a certain percentage of paths to the robot with the least current workload, thereby reducing the computational complexity by decreasing the number of paths allocated each time, and improving the efficiency and quality of path allocation. At the same time, compared with the direct segmentation methods, this algorithm can reduce the number of turns and arc ignition points, and improve the forming quality. The specific workflow of the “top k%” algorithm is shown in Figure 18.

**Figure 19:**
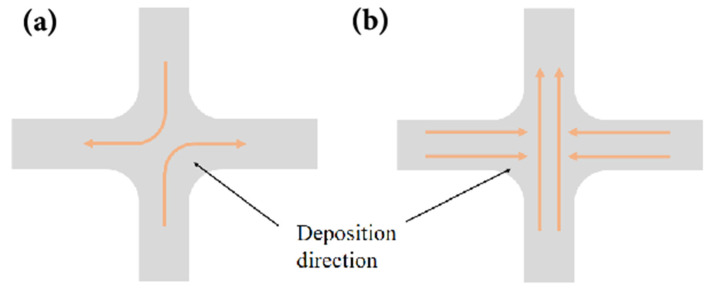
Typical deposition strategies for intersections: (**a**) path pattern of opposite angles; (**b**) one direct and two crossing methods.

**Figure 20:**
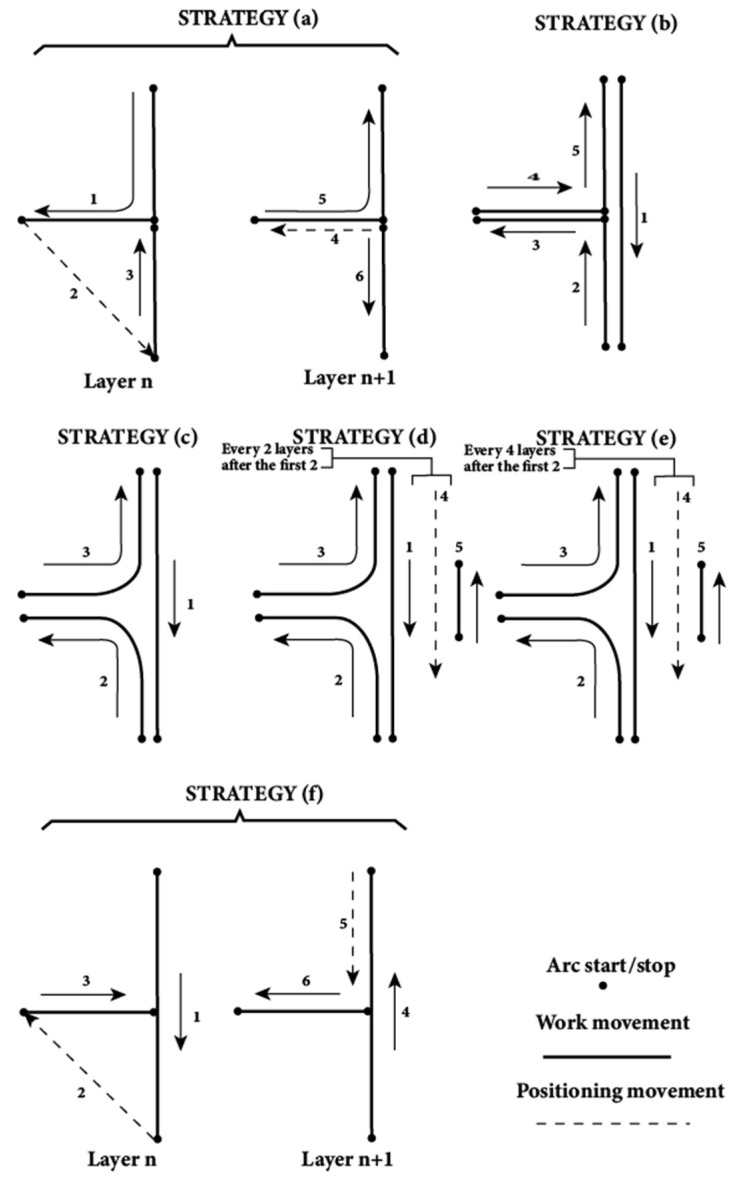
Schematic diagram of different deposition paths at T-shaped intersections in WA-DED process (In the figure, (**a**–**f**) represent six different types of paths designed for T-shaped intersection deposition, with the numbers indicating the deposition sequence) [171].

During the deposition process, encountering intersections or sharp-angle corners often leads to the formation of defects. There are two methods to avoid direct deposition of intersections: one is to deposit at opposite angles [169], as shown in Figure 19a, and the other is to first deposit a straight weld bead and then deposit branches on both sides of the straight weld bead, as shown in Figure 19b. The former can avoid the intersection by changing the deposition direction, and the path planning algorithm based on it has high universality and relatively simple. Therefore, Ding et al. [170] proposed a MAT-based planning algorithm capable of opposite angle deposition, thereby avoiding deposition at intersections. Venturini et al. [171] used six different types of paths design for T-shaped intersection deposition and selected the optimal deposition path (f) based on evaluation parameters such as surface quality, continuity, and stability of the deposition path, as shown in Figure 20. They also pointed out that rounded corners can be used instead of sharp corners as the inner angle of the T-shaped intersection, and if sharp corners are needed, milling operation is necessary. For deposition of parts with sharp corners or high curvature, most current research focuses on path planning to reduce or avoid direct deposition on these complex features, or relies on empirical adjustments of process parameters to improve forming quality [172].

## 5. Auxiliary Energy and Mechanical Fields

The predominant auxiliary fields integrated into WA-DED technology include magnetic, ultrasonic and mechanical fields. These fields act on the molten pool and the deposited layer during the additive manufacturing process. Collectively, the auxiliary fields have the benefits of improving the forming accuracy and surface quality of the parts, and reducing the residual stresses [173].

**Figure 21:**
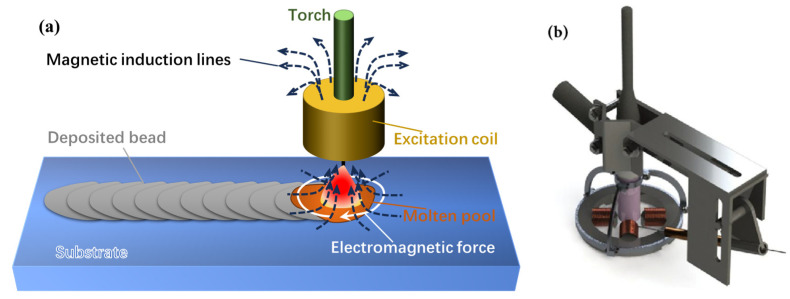
Schematic diagrams of the external magnetic field assisted WA-DED: (**a**) the longitudinal magnetic field; (**b**) the transverse magnetic fields [180].

When applying an auxiliary magnetic field, there are two primary methods, longitudinal and transverse, as shown in Figure 21, which have different effects on the deposition process. Applying a longitudinal magnetic field by setting coils around the welding torch can generate tangential electromagnetic forces, as shown in Figure 21a. The longitudinal magnetic field produces a stirring effect within the molten pool, which causes the molten metal to flow outwards. This can improve the forming accuracy and surface quality of the part [174]. Zhou et al. [175] successfully boosted the aspect ratio of weld beads and refined the surface quality of multilayer parts by utilizing an external longitudinal static magnetic field of 0.014 T. Similarly, Wang et al. [176] confirmed this finding, emphasizing the direct correlation between magnetic field intensity and excitation current. Their research revealed that, in the presence of a longitudinal magnetic field, adjusting the excitation current to 25 A led to an increase in the aspect ratio of weld beads, producing broader and flatter cross-sections. This enhancement significantly improved lap precision and deposition layer smoothness. Nevertheless, a further increase in the excitation current to 30 A resulted in a decline in surface smoothness.

Additionally, the longitudinal magnetic field can compress or expand the arc, and even cause the arc to rotate along the axis of the welding wire, thereby increasing or decreasing the arc energy density to obtain different deposition layer sizes [177,178]. When the coil is placed horizontally, with the coil axis perpendicular to the torch, a transverse magnetic field can be applied, as shown in Figure 21b. This magnetic field can deflect the arc backward toward the rear of molten pool, causing a unidirectional forced convection that drives the metallic fluid and heat to move toward the rear of the molten pool [179]. The influence of the magnetic field on arc and molten pool flow has been extensively and intensively studied in welding processes. But further research is required to understand its impact on the forming accuracy and surface quality of WA-DED parts.

In the arc welding process, it has been found that the acoustic radiation force of ultrasound has the effect of compressing the arc, changing the behavior of the molten droplets [181]. It can also increase the penetration of the molten pool, and made the weld bead more uniform and consistent [182,183]. Current studies have found that applying ultrasound in WA-DED technology can reduce the porosity and residual stress of parts, potentially decreasing or even eliminating residual tensile stress [184]. Research indicates that the introduction of a 25 kHz ultrasonic vibration frequency leads to a reduction in porosity. However, as the ultrasonic frequency rises from 25 kHz to 41 kHz, porosity gradually increases. At a frequency of 25 kHz, the acoustic streaming effect of ultrasound plays a pivotal role in determining porosity. By enhancing mixing and stirring within the molten pool, this effect boosts fluidity, enabling gas bubbles to ascend and escape prior to solidification, thereby significantly lowering porosity. Conversely, as the ultrasonic frequency escalates from 25 kHz to 41 kHz, the cavitation effect takes precedence in influencing porosity. Yet, the cavitation thresholds—the minimal ultrasonic energy or intensity necessary to induce cavitation—increases with frequency, resulting in reduced cavitation, more trapped gas in the molten pool, and consequently, elevated porosity [185].

**Figure 22:**
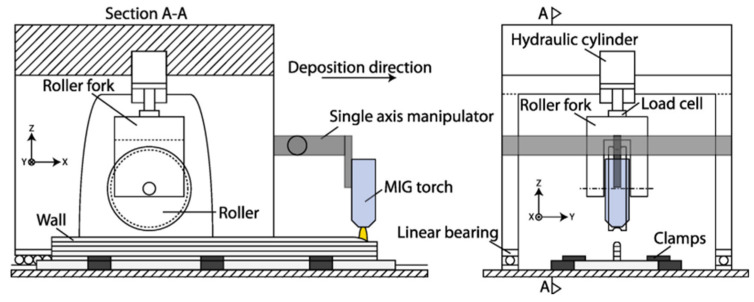
Schematic diagram of a typical rolling-assisted WA-DED technology [187].

**Figure 23:**
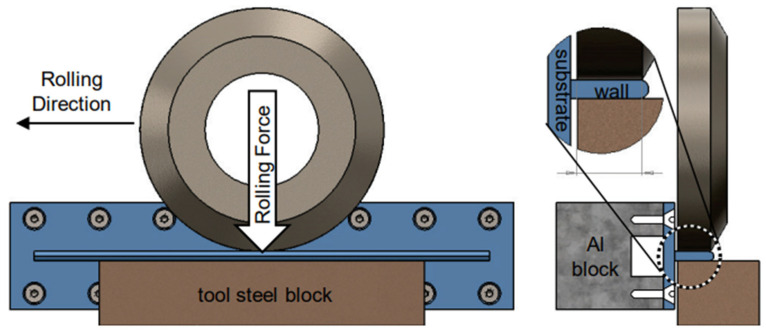
Schematic of side rolling for wall-shaped WA-DED parts [191].

In terms of applying auxiliary mechanical fields, research on inter-layer rolling during the deposition process is quite extensive. The inter-layer rolling technology was initially developed to eliminate residual stresses in deposited parts, thus to prevent deformation or cracking [186]. Some researchers have conducted cold rolling after the deposited layers cool to room temperature. For instance, Colegrove et al. [187] found when cold rolling was performed perpendicular to the substrate and along the deposition direction, it introduced compensatory plastic deformation into the deposition layer, thus counteracting residual stresses and deformation within the deposition layer. Besides, they used a roller with grooves to limit the lateral expansion of the deposition layer. Figure 22 shows this rolling-assisted WA-DED system. Similarly, Martina et al. [188] found that cold rolling treatment reduced residual stresses at the interface between the deposition layer and the substrate by 60% and deformation by more than half. However, cold rolling requires a longer interlayer cooling time, which can result in lower deposition efficiency. Some researchers have conducted hot rolling treatments on the deposited layers, such as Xie et al. [189] and Zhang et al. [190], who improved part deformation through hot rolling technology. However, some studies indicate that applying rolling perpendicular to the substrate along the deposition direction has limited effects on the residual stress and deformation of deposited components, as most deformation occurs perpendicular to the deposition direction. Honnige et al. [191] developed a side rolling technique, as shown in Figure 23, and pointed out that side rolling has the potential to completely eliminate the longitudinal residual stress and deformation of deposited parts.

The improvement of forming accuracy of WA-DED parts through rolling technology is significant, but it is difficult to roll deposited parts with corners or intricate features, which may potentially increase the complexity of path planning [192]. However, the increased equipment complexity associated with inter-layer rolling during the deposition will pose resistance to the widespread application of this technology. Hönnige et al. [193] proposed using a new type of machine hammer peening for greater design flexibility as a supplement or alternative to rolling in assisting the deposition process. Yang et al. [194] conducted inter-layer hammering at an inter-layer temperature of 800 °C and found that the maximum longitudinal and transverse residual stresses in the deposited wall structure were reduced by 20.7 and 273.9 MPa, respectively. Liang et al. [195] applied ultrasonic impact during the deposition process, which decreased the average residual stress on the surface of the part by 22.3% and the average deformation by about 20%. However, the ultrasonic amplitude should not be too large, or it may cause cracking of the deposited layer [196]. Furthermore, processes such as laser shock peening [197] and shot peening [198] can induce a small amount of plastic deformation on the surface of parts, converting tensile residual stress into compressive residual stress, but their effective depth is limited. So, these methods may be suitable for stress relief treatments that target only the part surface or each newly deposited bead. The integration of WA-DED technology with auxiliary fields is an emerging trend. [199]. The flexibility of installing lighter, more adaptable auxiliary field devices on the deposition torch will be a topic worth investigating.

Researchers also explore hybrid multi-heat source methods suitable for WA-DED process, that is, the use of two or more basic arc welding technologies or other heat sources (laser, flame, etc.) for deposition. These technologies can adjust the heat input, stabilize the arc, change the behavior of metal transfer (droplet), and increase the molten pool penetration, etc., thereby indirectly affecting the formation of the WA-DED parts. Additionally, they can directly improve surface quality and forming accuracy by remelting the top surface of each layer with the heat source positioned behind, which plays an important role in enhancing microstructure and mechanical properties of deposited parts [200,201].

## 6. Hybrid Additive and Subtractive Processing

**Figure 24:**
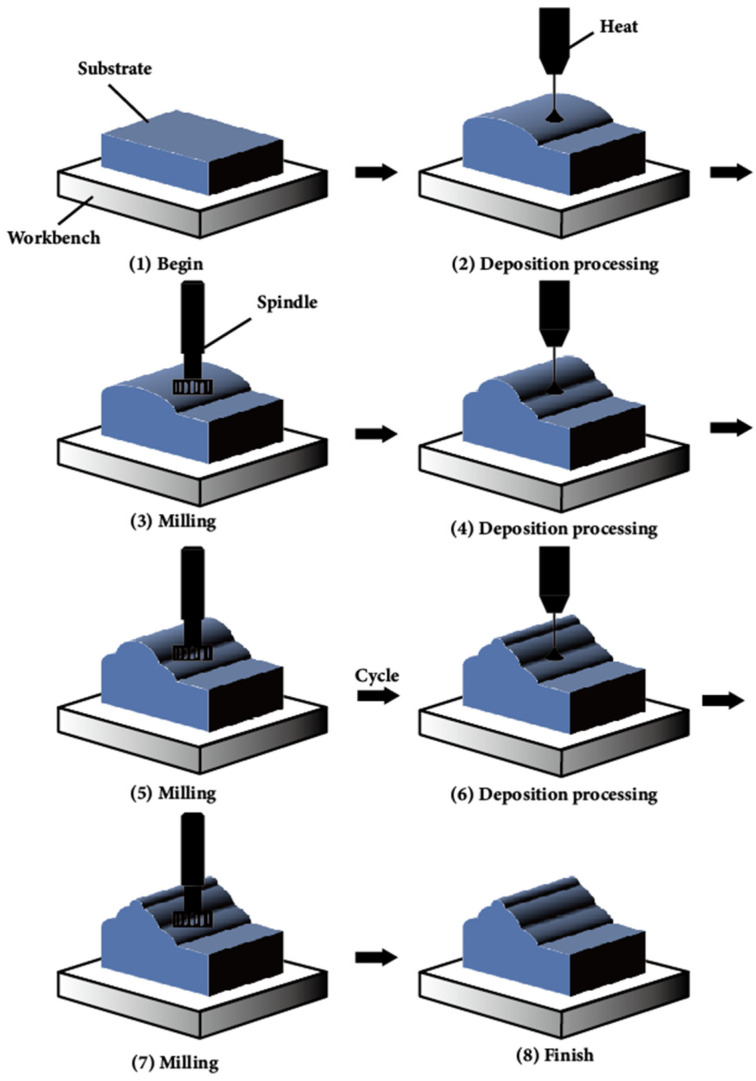
Schematic diagram of a typical hybrid additive and subtractive processing.

Additively manufactured metal parts often need to be post-treated by means of subtractive manufacturing technologies, such as machining, in order to obtain a flat and smooth surface. Additionally, machining can be used to rework features like corners, curves, and holes in the deposited parts, compensating for the difficulties and low accuracy associated with depositing these features. The industry has developed the concept of hybrid additive and subtractive processing, leveraging the rapid prototyping capabilities of additive manufacturing to realize the principle of “design equals manufacturing”, thereby improving material utilization and enabling the one-time production of complex-shaped and large-scale parts. Meanwhile, it capitalizes on the superior dimensional accuracy of subtractive manufacturing to compensate for the limitations in forming accuracy and surface quality inherent in additive manufacturing, to achieve a synergy where the advantages of subtractive and additive manufacturing complement one another [200,202]. On this foundation, researchers have tried to integrate machining tools with an additive manufacturing equipment, or integrate heat sources such as the electric arcs into a CNC machining center to achieve simultaneous additive and subtractive processing at a single workstation [203]—during additive manufacturing process, tools like mills can be used to machine the already solidified deposition layers—or it allows for a continuous process of additive followed by subtractive manufacturing in every layer’s manufacturing. This led to the development of the concept of integrated additive-subtractive manufacturing method. The typical process flow is shown in Figure 24. In terms of integration, how to perform tool change is the primary issue to consider. Li et al. [204] developed a straightforward hybrid manufacturing platform that comprises two robotic arms: one for milling and the other for deposition. Both arms share the same coordinate origin, simplifying the complex calibration process for the robotic arms. Zhang et al. [205] manufactured a mid-convex and hollow metal part that is not easily machinable by designing different programs for the tool station switching between milling and deposition. Li et al. [206] employed a 6-DOF industrial robotic arm, equipped with an interchangeable milling head, for integrated additive and subtractive manufacturing. They mapped out the milling features and paths, establishing parameters such as milling sequence, depth, speed, and others. Subsequently, they generated milling path codes that directed the robotic arm to execute milling operations precisely along the pre-planned routes. In hybrid additive and subtractive manufacturing, in addition to the transition between cutting tools, how to reasonably slice the workpiece is also very important. Ren et al. [207] used an equidistant slicing strategy for path planning and verified the part repair capability of hybrid additive and subtractive manufacturing technology. The equidistant slicing strategy only requires listing the slicing layers along the build direction, which is relatively straightforward and easy to automate. However, this method can lead to discontinuities between layers. Chang et al. [208] proposed a slicing strategy based on surface splitting, which under the premise of preventing collisions between the tool and the workpiece, can merge multiple surfaces into the same layer for processing. This reduces the number of slicing layers needed to construct a part and simplifies the calculation and planning process. For parts with complex shapes, experienced designers are often needed to correct the process. In the future, there is a need to develop smarter and more convenient slicing strategies for additive-subtractive hybrid manufacturing to improve the technology’s production efficiency and practicality [209]. Moreover, the design of the overall process planning is crucial, which encompasses the aforementioned identification, segmentation of part models into production machining sequences, and additionally, the design of parameters for both additive and subtractive manufacturing processes. Chen et al. [210] detailed the steps for fabricating parts after integrating additive manufacturing into a computer numerical control (CNC) machining center. This process involves reserving machining allowances on surfaces with tight tolerance requirements during deposition, and individually calculating and setting angles, tools, depths, and tool-inclusive boundaries for critical surfaces of the deposited part through process planning during milling. They also highlighted that the current configuration of three-axis machining has limitations when depositing parts with complex features, especially those that are not parallel or perpendicular to the machine axis.

Milling, as a quintessential mechanical machining process, can achieve precision to the micrometer level. The combination of milling and WA-DE technology is now an extensively researched theme [211]. Song et al. [212] successfully used this hybrid technology to improve the dimensional accuracy of the deposited parts from ±0.5 mm to 20 μm, and further to reduce the surface roughness to 2 μm. Akula et al. [213] combined CNC milling with WA-DED technology and used a customized software for milling the sides of the deposited metal layers from the top to the bottom, thereby achieving the desired forming dimensions and precision. Zhang et al. [214] performed milling after each layer of deposition and found that a milling depth within the range of 0.4–1.2 mm reduced the side surface roughness by 22.9% compared to the deposited parts without milling. However, when the milling depth was increased to 1.6 mm, the side surface roughness was 71.5% higher than that of the purely deposited parts. This is due to the slower flow of molten metal across the milled plane, which was nearly as wide as the layer’s cross-section, causing the molten metal to solidify before reaching the edge of the preceding layer, thereby leading to a decrease in the side surface quality. Therefore, the milling depth should be controlled within a certain range according to the actual width of the deposited layer to help improve the forming accuracy and surface quality. Han et al. [215] employed a new technology of ultrasonic-assisted milling to machine the deposited layer. The high-frequency vibration induced by ultrasound reduced the cutting force and mitigated the impact and friction during the cutting process, achieving higher surface accuracy of the part than conventional milling.

**Figure 25:**
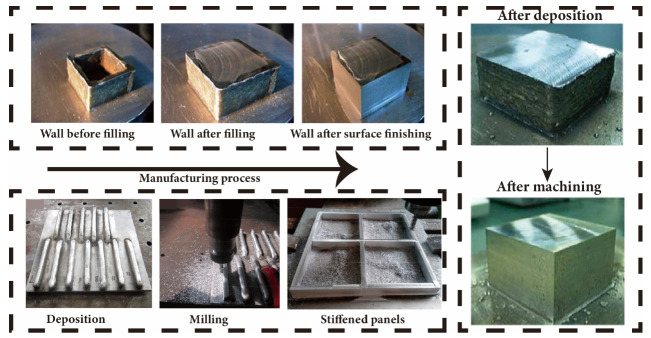
Example of hybrid manufacturing parts [212,217,218].

Research has demonstrated that milling can dramatically slash average surface residual stress by 93%. This phenomenon is attributed to the alternating nature of residual stress during the milling process. Specifically, when molten droplets arrive on previously deposited layers, the rapid cooling of these layers, constrained by the surrounding material, leads to the generation of elastic tensile stress. Consequently, tensile stress arises during the cooling phase. As milling progresses, the heat it generates reheats the deposited layers, effectively releasing the tensile stress [216]. Furthermore, the friction between the cutter and the milling surface, known as the burnishing effect, results in milling-induced compressive stress. This not only diminishes surface residual stress but also neutralizes the maximum internal stress point by counteracting the initial residual tensile stress. The remaining residual stress can be further reduced through heat treatment. Figure 25 shows a component produced through the combined use of hybrid additive and subtractive manufacturing, showcasing the impact of milling after additive manufacturing [212,217]. Li et al. [218] leveraged hybrid additive and subtractive manufacturing to fabricate stiffened panels, which are extensively utilized in sectors such as aviation, aerospace, and automotive. Their findings revealed that the use of a tandem GMA-DED process integrated with milling significantly enhances material utilization by 57% and boosts productivity by 32%, compared to conventional milling techniques. The potential for development of hybrid additive and subtractive manufacturing technology in the manufacturing field is evident.

## 7. Conclusions

With the increasing demand for high-performance, large-scale, integral metal parts in the manufacturing industry, the advantages of WA-DED technology are becoming increasingly prominent. Enhancing its shape-control capabilities is essential for the broader application of this process. This paper systematically introduces a variety of shape control strategies for WA-DED, which provides a reference for ensuring the forming accuracy and surface quality of WA-DED parts, and the main conclusions are as follows:

1. The WA-DED process based on different types of arc welding technology has distinct process windows. The comprehensive effects of electrical parameters can be studied using the variable of heat input. Excessive heat input can cause thermal stress and deformation in parts. When the wire feeding process is decoupled from the arc, such as in GTA-based and PA-based DED processes, the wire feed rate significantly affects deposition layer height, while the deposition current largely influences layer width.
2. When depositing parts with large inclination angles and overhanging structural features, the welding torch angle can be adjusted to prevent the molten pool from sagging. The application of auxiliary energy fields to alter the force state of the droplet and molten pool can also improve the forming accuracy of the deposited weld bead and the whole additively manufactured part.
3. Regulating droplet size and transfer frequency can directly affect the forming accuracy and surface quality of WA-DED parts. This influence is often realized by altering the droplet’s interaction with the molten pool. Additionally, GTA-based DED with side wire feeding may lead to deposition deviation, necessitating optimization of wire feeding control methods and regulation of droplet landing locations.
4. Controlling heat input, preheating the substrate, and forced cooling can all regulate inter-layer temperature. Essentially, these methods are employed to reduce the thermal gradient in the additively manufactured part, thereby reducing deformation caused by thermal stress. Moreover, the design of substrate thickness and structure can alter the substrate’s heat dissipation characteristics and restraint effects, meeting the requirements for additive manufacturing with low residual stress and deformation.
5. Different deposition paths can lead to variations in the temperature distribution and stress state within the deposited parts, thereby affecting deformation. An optimal path planning strategy should aim to minimize the number of turnarounds and arc initiation points. In multi-layer additive manufacturing processes, the reverse directional deposition is frequently applied. The design of spacing between adjacent additive weld beads should consider bead shift caused by surface tension.
6. The application of different auxiliary fields in WA-DED is a current research hotspot. Longitudinal magnetic field helps increase the width-to-height ratio of deposited beads, improving lap joint morphology and the flatness of deposited layers. The application of ultrasound in WA-DED process can reduce or even eliminate residual tensile stress. Rolling combined with WA-DED can improve the forming accuracy of deposited layers, but it is more challenging to apply to parts with corners or tortuous features. Laser shock peening and shot peening are suitable for stress relief treatment on the surface and newly deposited additive weld beads. More lightweight, flexible, and accessible field-assisted WA-DED technologies have a promising future.
7. In the field of high-precision manufacturing, subtractive post-processing such as machining is indispensable. Hybrid additive and subtractive processing and even integrated additive-subtractive manufacturing method will be popular research directions.

## Figures and Tables

**Table 1:** Summary of measures to mitigate forming defects in WA-DED.

Problems	Mitigations
Problem 1	Control of interlayer temperature can be achieved by preheating the substrate, real-time temperature detection and control, and adding external auxiliary cooling equipment for forced cooling to reduce the temperature gradient during the deposition process. Detailed content will be elaborated in Section 3.5 of this article. Designing the thickness and structure of the substrate can alter its heat dissipation characteristics, thereby reducing thermal stress. Detailed content will be elaborated in Section 3.6 of this article. The choice of deposition path, such as using reciprocating deposition rather than unidirectional deposition in most cases, can help reduce the temperature gradient. Detailed content will be elaborated in Section 4 of the article. Mechanical field assistance in rolling the part can alter its stress state. Detailed content will be elaborated in Section 5 of this article.
Problem 2	Hybrid additive and subtractive manufacturing (this also applies to the third issue). Detailed content will be elaborated in Section 5 of this article. Adding auxiliary energy fields (ultrasonic field, magnetic field) to increase the constraint on the molten pool. (This also applies to the third issue). Detailed content will be elaborated in Section 6 of this article.
Problem 3	Selecting appropriate electrical parameters, wire feed speed, and travel speed to ensure that the heat input remains within a reasonable range, allowing the molten pool to solidify in a timely manner. Detailed content will be elaborated in Section 3.1 of this article. Adjusting the torch angle to increase the constraint when depositing parts with larger inclination angles. Detailed content will be elaborated in Section 3.3 of this article. Controlling the droplet transfer behavior by adjusting the electrical parameters, as generally, free transition results in greater impact force on the molten pool. Detailed content will be elaborated in Section 3.4 of this article.

**Table 2:** Four main types of models for additive weld bead overlap.

Schematic Diagram of the Additive Weld Bead Overlapping Models	Characteristics of the Models
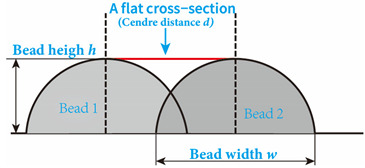	Flat-top overlapping model (FOM), utilizing a straight line to connect the vertices of the adjacent weld beads. The optimal center distance is 0.6366 *w* [157].
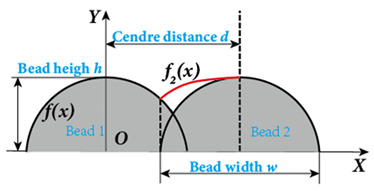	The improved FOM model, utilizing the parabolic function *f*_2_(x) to fit the overlapping area. The optimal center distance is 0.715 *w* [158].
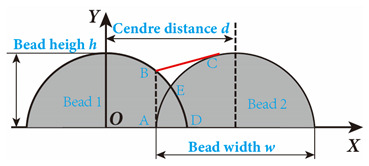	Tangent overlapping model (TOM), with the definition of the critical valley (the area surrounded by points B, C, and E) introduced. (In the figure, point A is the leftmost endpoint of bead 2. And point D is the rightmost endpoint of bead 1. A line parallel to the Y-axis passing through point A intersects bead 1 at point B. Line BC is tangent to bead 2, and point E is the intersection point of the contours of bead 1 and bead 2.) The optimal center distance is 0.738 *w* [159].
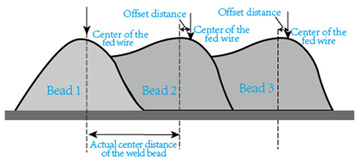	Improved overlapping model, taking into account in advance the offset distance of weld bead caused by the actual physical process (surface tension), performing deposition of non-isometric movement of the welding torch [160].
